# Inhibition of Sirt2 Alleviates Fibroblasts Activation and Pulmonary Fibrosis *via* Smad2/3 Pathway

**DOI:** 10.3389/fphar.2021.756131

**Published:** 2021-12-01

**Authors:** Hui Gong, Chenyi Zheng, Xing Lyu, Lini Dong, Shengyu Tan, Xiangyu Zhang

**Affiliations:** ^1^ Department of Geriatrics, The Second Xiangya Hospital, Central South University, Changsha, China; ^2^ Laboratory of Clinical Medicine, The Second Xiangya Hospital, Central South University, Changsha, China

**Keywords:** Sirtuin2, pulmonary fibrosis, fibroblast activation, AGK2, Smad2/3

## Abstract

Idiopathic pulmonary fibrosis (IPF) is a fatal disease with unknown cause and limited treatment options. Its mechanism needs to be further explored. Sirtuin2 (Sirt2), a nicotinamide adenine dinucleotide (NAD)-dependent deacetylase, has been proved to be involved in the fibrosis and inflammation in the liver, kidney and heart. In this study, we aimed to evaluate the role of Sirt2 in pulmonary fibrosis. We found that Sirt2 expression was upregulated in transforming growth factor-*β*1 (TGF-*β*1) treated human embryonic lung fibroblasts. Sirt2 inhibitor AGK2 or the knockdown of Sirt2 expression by targeting small interfering RNA (siRNA) suppressed the fibrogenic gene *α*-SMA and Fibronectin expression in TGF-*β*1 treated fibroblasts and primary lung fibroblasts derived from patients with IPF. In addition, Sirt2 inhibition suppresses the phosphorylation of Smad2/3. Co-immunoprecipitation (Co-IP) showed that there is interaction between Sirt2 and Smad3 in the TGF-*β*1 treated lung fibroblasts. In bleomycin-induced pulmonary fibrosis in mice, AGK2 treatment significantly mitigated the degree of fibrosis and decreased the phosphorylation of Smad2/3. These data suggest that Sirt2 may participate in the development of IPF *via* regulating the Smad2/3 pathway. Inhibition of Sirt2 would provide a novel therapeutic strategy for this disease.

## Introduction

Idiopathic pulmonary fibrosis (IPF) is a devastating disease with increasing morbidity, and the median survival of the patients is only 3–5 years after diagnosis (Chanda et al., 2019). There is no effective treatment for this disease (Noble et al., 2012). The pathogenesis of IPF remains unclear. Aberrant activation and differentiation of fibroblasts to myofibroblasts plays a critical role in the development of this disease (Wynn and Ramalingam, 2012; Selman and Pardo, 2014; Meiners et al., 2015). Myofibroblast differentiation is induced by various cytokines and chemokines (Ballester et al., 2019), among these, transforming growth factor-*β*1 (TGF-*β*1) is a well-documented mediator (Meng et al., 2016; Morikawa et al., 2016). Myofibroblasts are characterized by the expression of *α*-smooth muscle actin (*α*-SMA), excessive accumulation of extracellular matrix (ECM) components including Fibronectin and collagen, which would form fibrotic scars and eventually lead to the loss of tissue function (Wynn, 2008). Understanding the molecular mechanisms of lung fibroblasts activation is important for developing new anti-fibrotic agents.

Accumulating evidence supports the role of epigenetic alterations including histone acetylation in the pathogenesis of IPF (O'Reilly, 2017; Wallner et al., 2020; Jones et al., 2019). Histone acetylation is regulated by histone deacetylases (HDACs) and histone acetylases (HATs) (Drazic et al., 2016). Sirtuins are Class III HDACs that are nicotinamide adenine dinucleotide (NAD^+^) dependent deacetylase (Imai and Guarente, 2014), including seven members (Sirtuin 1–7) (Gomes et al., 2019). The cytosol member Sirtuin 2 (Sirt2) is widely expressed in almost all mammalian organs. Previous studies suggest that Sirt2 is involved in inflammatory response and fibrosis progress in different organs, including kidney, heart and liver, however, its role is controversial (Ponnusamy et al., 2014; Arteaga et al., 2016; Tang et al., 2017). For instance, Sirt2 acts as a cardio-protective deacetylase in aging-related and angiotensin II (Ang II)-induced cardiac fibrosis and hypertrophy, and loss of Sirt2 promotes these pathological changes (Tang et al., 2017). While in hepatic and renal fibrosis, Sirt2 demonstrated the pro-fibrogenesis characteristics, blocking Sirt2 inhibited the activation of hepatic stellate cells and renal interstitial fibroblasts, and suppressed hepatic and renal fibrosis (Ponnusamy et al., 2014; He et al., 2018). It is noteworthy that a study involving triple antigen induced-allergic eosinophilic asthma has proved a stimulatory role of Sirt2 on the recruitment of eosinophils. This indicates a pro-inflammatory effect of Sirt2 in pulmonary microenvironment (Lee et al., 2019). The role of Sirt2 in pulmonary fibrosis remains elusive.

In the current study, we evaluated the role of Sirt2 in TGF-*β*1 induced lung fibroblasts activation and bleomycin induced pulmonary fibrosis in mice. Our findings indicated for the first time that the expression of Sirt2 is increased in TGF-*β*1- activated lung fibroblasts and fibrotic lung tissues of mice induced by bleomycin. Sirt2 inhibition suppressed the fibrogenic gene *α*-SMA and Fibronectin expression in TGF-*β*1 treated lung fibroblasts and primary lung fibroblasts derived from patients with IPF. In addition, Sirt2 inhibition suppresses the phosphorylation of Smad2/3. Co-immunoprecipitation demonstrated the interaction between Sirt2 and Smad3 in lung fibroblasts. In animal model, inhibition of Sirt2 alleviated pulmonary fibrosis and reduced the phosphorylation of Smad2/3 induced by bleomycin.

## Materials and Methods

### Cell Culture and Treatment

The human embryonic lung fibroblasts (MRC-5) used in this study were purchased from the Chinese academy of sciences (Cat. no. GNHu41, Shanghai, China). Human primary IPF lung fibroblasts were purchased from The Global Bioresource Center (ATCC® CCL-134™, United States). In TGF-*β*1 concentration-dependent assay, when the MRC-5 reached 80% confluence, the growth medium was changed to serum free medium overnight; then the cells were treated with Recombinant human TGF-*β*1 (R&D Systems, Minneapolis, MN) at 0, 1, 2, 5, and 10 ng/ml for 24 h. In the time-dependent test, the cells were cultured for a period of 0, 3, 6, 12, 24, and 48 h at 2 ng/ml TGF-*β*1. In Sirt2 inhibition study, MRC-5 cells were treated with TGF-*β*1 at 2 ng/ml for 24 h, and then added 10 μM AGK2 (an inhibitor of Sirt2) (MCE, HY-100578) or vehicle control (dimethyl sulfoxide, a AGK2 solvent) for another 24 h in the presence of TGF-*β*1.

### RNA Extraction and Real-Time RT-PCR

RNA was extracted with a RNeasy® Mini kit (Qiagen GmbH, Hilden, Germany), and converted into cDNA using a Revert Aid First stand cDNA synthesis Kit (Thermo Scientific, United States). Real-time PCR was performed using a SYBR Green/qPCR Master Mix kit according to the manufacturer’s instructions (Thermo Scientific, United States). Real-time RT-PCR was performed in triplicate and normalized to GAPDH or *β*-actin with the ΔΔ*C*t method. Primers are listed in Table 1.

**TABLE 1:** Primers used in the real-time RT-PCR.

Gene name	Sequence
Sirt2	F: 5′-TGC​GGA​ACT​TAT​TCT​CCC​AGA-3′
	R: 5′-GAG​AGC​GAA​AGT​CGG​GGA​T-3′
Fibronectin	F: 5′-TCG​CTT​TGA​CTT​CAC​CAC​CAG-3′
	R: 5′-CCT​CGC​TCA​GTT​CGT​ACT​CCA​C-3′
*α*-SMA	F: 5′- CTA​TGA​GGG​CTA​TGC​CTT​GCC-3′
	R: 5′-GCT​CAG​CAG​TAG​TAA​CGA​AGG​A-3′
*β*-actin	F: 5′-CTG​TCC​CTG​TAT​GCC​TCT​G-3′
	R: 5′-ATG​TCA​CGC​ACG​ATT​TCC-3′
GAPDH	F: 5′-CCC​ATG​TTC​GTC​ATG​GGT​GT-3′
	R: 5′-TGG​TCA​TGA​GTC​CTT​CCA​CGA​TA-3′

### Western Blot Analysis

Whole cell lysates were collected with RIPA Lysis Buffer containing protease and phosphatase inhibitor mixture. The total protein concentration of the lysates was quantified using a Micro BCA Protein Assay Kit (Thermo Scientific, United States). The same amount of protein was electrophoresed on 10% SDS-PAGEs and Western immunoblotting was performed according to the manufacturer’s instructions. Immunoblots were imaged using an Amersham Biosciences 600 imager. Quantification of protein expression for all blots was performed using ImageJ software. Primary antibodies Sirt2 (1:1000; #9787), *α*-SMA (1:1000; #19245), Fibronectin (1:1000; #26836), phospho-Smad2 (1:1000; #3108), phospho-Smad3 (1:1000; #9520), Smad2/3 (1:1000; #8685), GAPDH (1:1000; #2118), *β*-actin (1:1000; #4970), and horseradish peroxidase-conjugated secondary antibody (1:5000; #7074) were all from Cell Signaling Technology.

### Immunofluorescence Staining

MRC-5 cells were cultured on coverslips as described previously with or without 10 μM AGK2 in the presence of TGF-*β*1 for 24 h. The cells were fixed with 4% paraformaldehyde for 15 min at room temperature, permeabilized with 0.1% Triton X-100 for 10 min, blocked with 10% normal goat serum and incubated with anti-Fibronectin (1:400) or anti-*α*-SMA (1:400) followed by Alexa Fluor 558 goat anti-rabbit secondary antibody (1:1000). Fluorescence images were collected on a fluorescence microscope.

### Small Interfering RNA Transfections

When MRC-5 cells and IPF lung fibroblasts grew to 70–80% confluence, the cells were transfected with negative control small interfering RNA (NC siRNA) or Sirt2 targeted siRNA (Sirt2 siRNA) (Santa Cruz Biotechnology, sc-40988, Inc. United States) using lipofectamine® 3000 (Invitrogen, Carlsbad, CA, United States) according to manufacturer’s instructions. After 24 h transfection, the medium was changed, and cells were incubated for another 24 h in the absence or presence of 2 ng/ml TGF-*β*1. The efficiency of transfection was evaluated by Sirt2 mRNA and protein expression using quantitative real-time RT-PCR and Western blot.

### Co-Immunoprecipitation

Co-IP was carried out with IP/Co-IP Kit (88,804; Thermo Fisher Scientific, United States). Pierce protein A/G-Agarose beads were washed using 100 μl antibody binding and washing buffer to wash. Beads were collected and gently rotated with rabbit anti-Sirt2 (1:20, ab211033, Abcam) or rabbit IgG (1:20, ab6715, Abcam) antibodies for 10 min after supernatant was removed. Subsequently, incubate the antibody-beads complex with 400 μg total protein from hypotonic lysis buffer for 5 min. The supernatant was removed and the antibody-protein-beads complex was washed 3 times using washing buffer. The supernatant was removed again and the antibody-protein beads complex was gently resuspended with 100 μl elution buffer for 2 min. The sample was separated and subjected to Western blot analysis.

### Experimental Mice Model of Pulmonary Fibrosis

Animal studies were approved by the Animal Ethics Committee of the Second Xiangya Hospital, Animal Center of Central South University (Approval No. 2021026). 6-8-week-old healthy C57BL/6 mice (male, 20–25 g) were randomly divided into three experimental groups: control group (n = 6, with saline treatment), bleomycin (BLM) group (n = 6, with BLM treatment), and BLM + AGK2 group (n = 6, with BLM/AGK2 co-treatment). A single dose of bleomycin sulfate at 1.5 U/kg body weight was conveyed *via* transtracheal injection. AGK2 in dimethyl sulfoxide solution was administered via daily intraperitoneal injection for successive 7 days at 50 mg/kg, starting at day 14 post-bleomycin injury. Mice were sacrificed on day 21 post bleomycin injury, and the lung tissues were prepared for Western blot and histology.

### Histological Staining and Immunohistochemical Staining

Lung tissues were fixed with 4% paraformaldehyde for 24 h and underwent dehydration by alcohol of different concentration. Tissues were embedded into paraffin and placed at room temperature for 24 h, then cut into 5 μm sections. Haematoxylin-eosin (HE) staining kit (cat. no. C0109; Beyotime) and Masson staining kit (cat. no. C0215; Beyotime) were used to determine the degree of alveolitis and fibrosis withSzapiel’s method (Szapiel et al., 1979). Images were captured under a microscope (BA210T; Motic). For IHC, the sections received antigen retrieval in citrate buffer at 95°C for 15 min, then blocked with 0.5% BSA-PBS containing 10% goat serum for 1h, and finally incubated with anti-Fibronectin (1:100; 66042-1-IG), anti-*α*-SMA (1:200; 55135-1-AP), or anti-Sirt2 (1:100; 19655-1-AP) antibody overnight. The density of positive areas was measured using Image-Pro Plus 6.0 software.

### Quantification and Statistical Analysis

GraphPad Prism version 7.0 software was used for graph preparation and data analysis. Densitometric analysis was performed by ImageJ software. All data were calculated as the means ± standard deviation (SD) based on at least three independent experiments. The significance of differences was analyzed using Student’s t test or one-way ANOVA. A *p*-value of less than 0.05 was statistically significant.

## Results

### Sirt2 Expression Is Increased in TGF-*β*1 Treated Lung Fibroblasts

**FIGURE 1:**
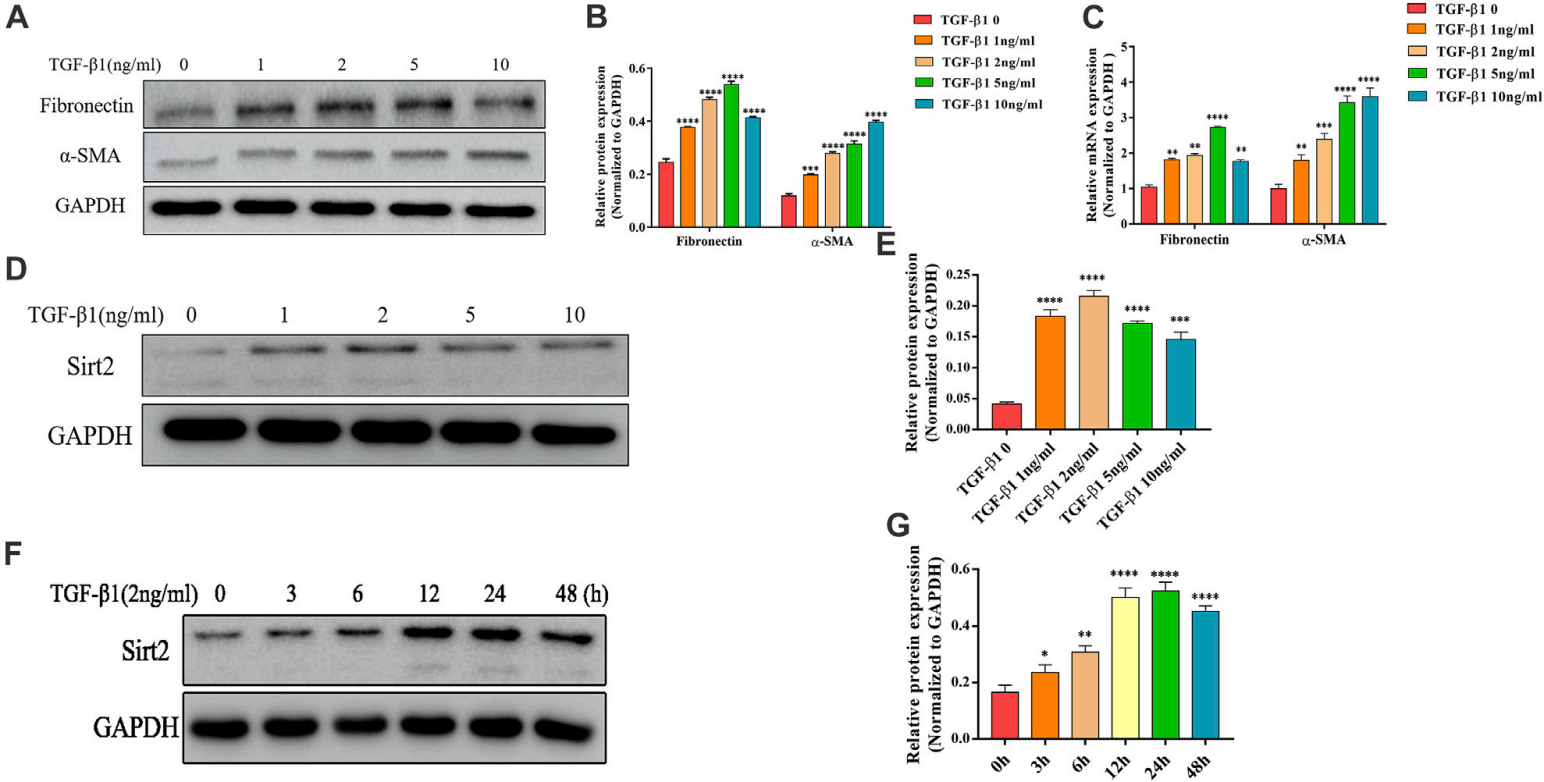
Sirt2 expression is increased in TGF-*β*1 stimulated lung fibroblasts. **(A,D)** Expression of Fibronectin, *α*-SMA and Sirt2 proteins detected by Western blot in MRC-5 cells treated with 0 (control), 1, 2, 5, and 10 ng/ml TGF-*β*1 for 24 h **(B,E)** Densitometric analyses of the Western blot in **(A,D)**. **(C)** Expression of Fibronectin and *α*-SMA mRNA detected by real-time RT-PCR in MRC-5 cells treated with 0 (control), 1, 2, 5, and 10 ng/ml TGF-*β*1 for 24 h. **(F)** Expression of Sirt2 protein detected by Western blot in MRC-5 after 2 ng/ml TGF-*β*1 exposure for 0, 3, 6, 12, 24, and 48 h. **(G)** Densitometric analyses of the Western blot in **(F)**. GAPDH was used as a loading control. The bars indicated mean ± SD of three separate experiments. *p <0.05, **p <0.01, ***p <0.001, ****p <0.0001 compared to the control.

Lung fibroblasts activation and differentiation is critical for the development of pulmonary fibrosis. Since TGF-*β*1 is a well-documented pro-fibrogenic cytokine in the progression of IPF, we used TGF-*β*1 as a fibroblast activator in this study. As shown in Figures 1A–C, after exposure to different concentrations of TGF-*β*1 (1, 2, 5 and 10 ng/ml for 24 h), the protein and mRNA expression of fibrogenic genes Fibronectin and *α*-SMA were increased significantly in MRC-5 cells. Next, in order to determine whether Sirt2 plays a role in pulmonary fibrosis, Sirt2 expression was examined in MRC-5 cells treated with the same concentration of TGF-*β*1 as described above (Figures 1A–C). As shown in Figures 1D,E, the protein expression of Sirt2 was elevated at different concentrations of TGF-*β*1 treatment, with a peak level at 2 ng/ml treatment. In order to determine the time course of TGF-*β*1 regulating Sirt2 expression, the cells were treated with 2 ng/ml TGF-*β*1 for different time periods and the results showed that Sirt2 expression was significantly increased at 3, 6, 12, 24, and 48 h, and the level peaked at 24 h after TGF-*β*1 stimulation (Figures 1F,G). The results suggested that Sirt2 may play a role in the process of lung fibroblasts activation.

### AGK2 Attenuates TGF-*β*1-Induced Lung Fibroblasts Activation

**FIGURE 2:**
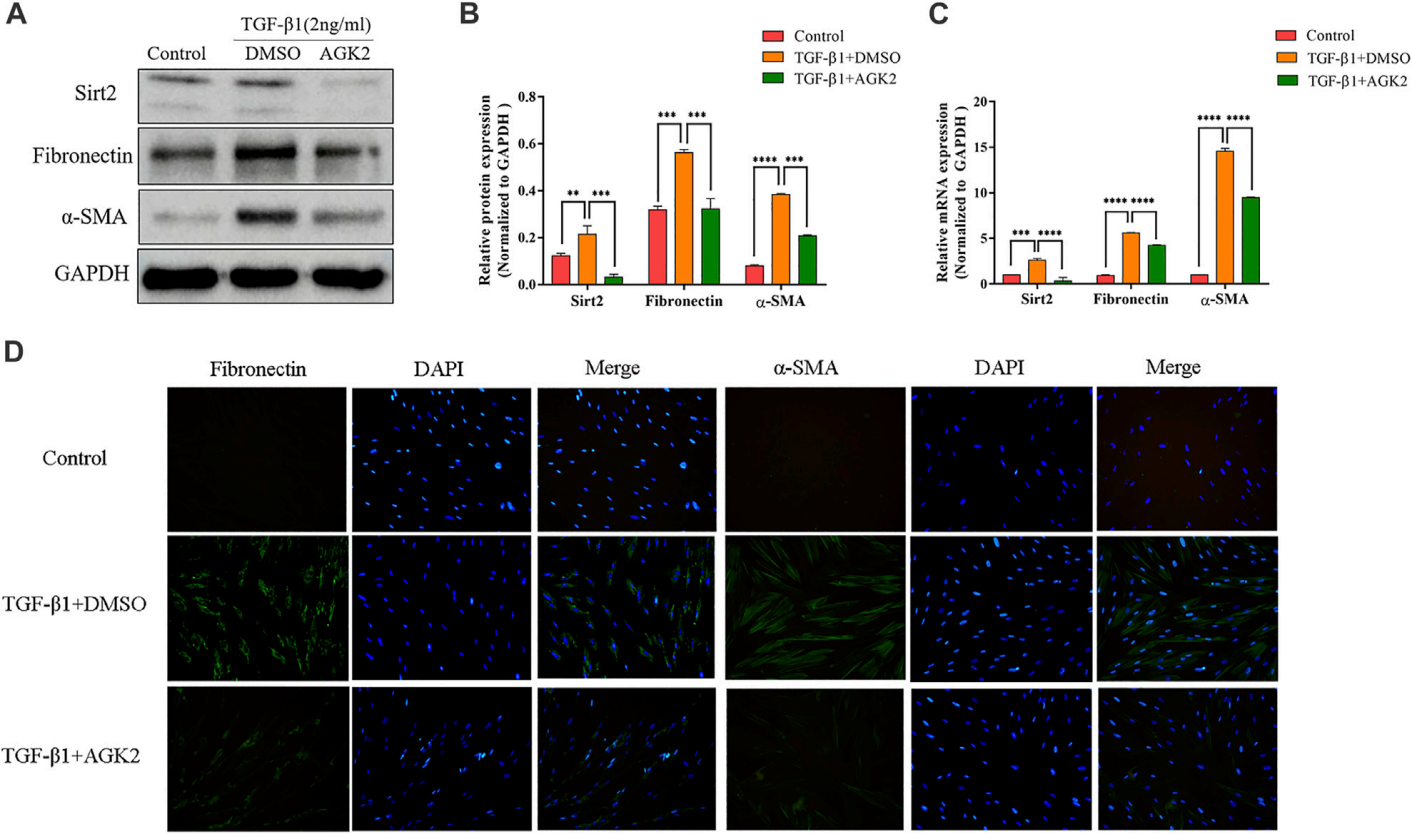
AGK2 decreases fibrogenic gene expression in TGF-*β*1-induced lung fibroblasts activation. **(A,C)** Protein and mRNA expression of Sirt2, Fibronectin and *α*-SMA proteins by Western blot and real-time RT-PCR in MRC-5 cells treated with 2 ng/ml TGF-*β*1 for 24 h and then added 10 μM AGK2 or DMSO for another 24 h in the presence of TGF-*β*1. GAPDH was used as loading control. **(B)** Densitometric analyses of the Western blot in **(A)**. **(D)** Fibronectin and *α*-SMA was strongly expressed in response to TGF-*β*1, and AGK2 decreased the expression by immunofluorescence staining. Green means Fibronectin and *α*-SMA staining; Blue means DAPI. The bars indicated mean ± SD of three separate experiments. DMSO, dimethyl sulfoxide; **p <0.01, ***p <0.001, ****p <0.0001 compared to the TGF-*β*1+DMSO group.

**FIGURE 3:**
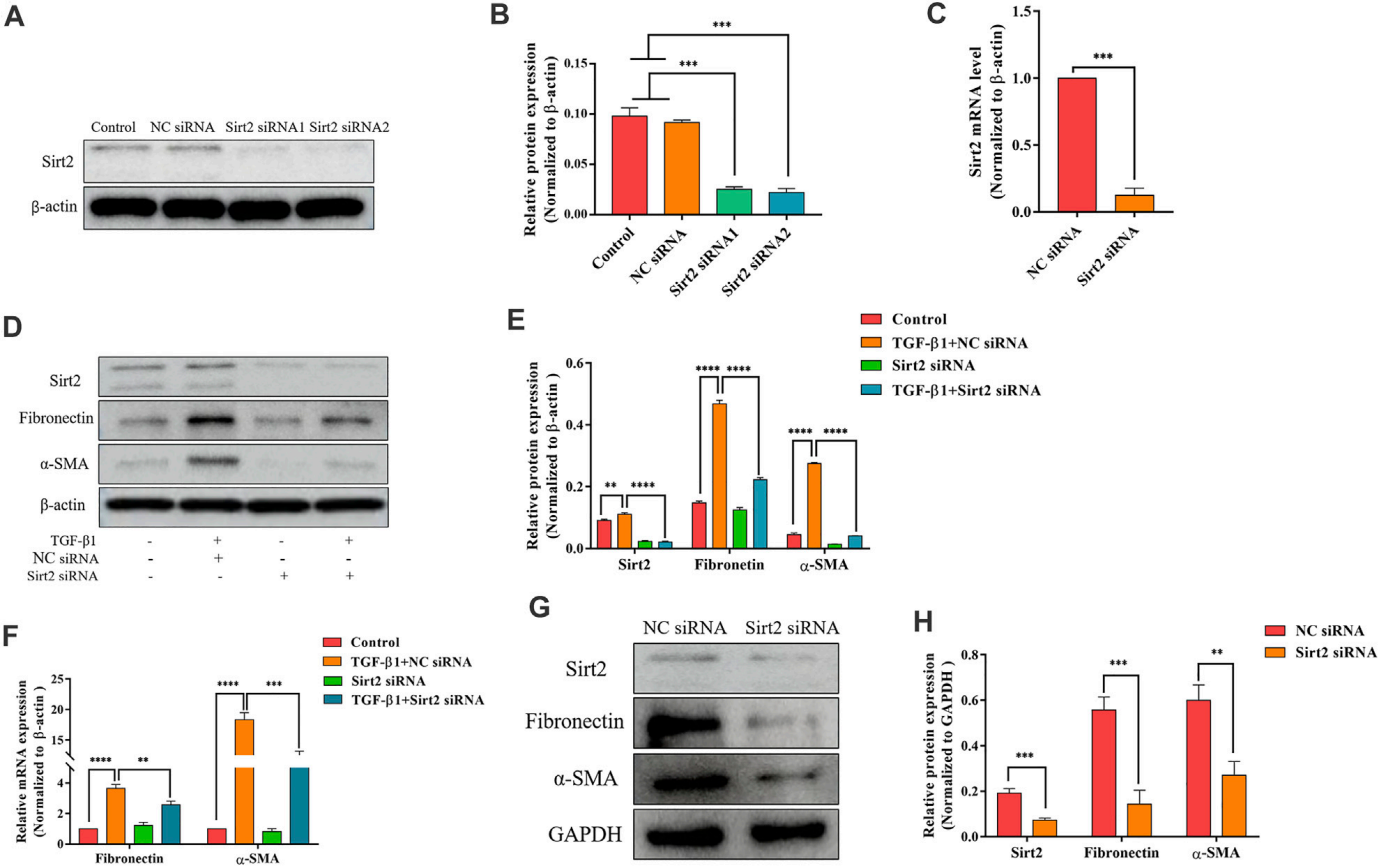
Silencing Sirt2 inhibits fibrogenic gene expression in TGF-*β*1-treated lung fibroblasts and IPF lung fibroblasts. **(A,C)** MRC-5 cells were transfected with NC siRNA or Sirt2 siRNA for 24 h, Sirt2 siRNA successfully downregulated Sirt2 protein and mRNA expression detected by Western blot and real-time RT-PCR. **(B)** Densitometric analyses of the Western blot in **(A)**. **(D,F)** Protein and mRNA expression of Sirt2, Fibronectin, and *α*-SMA by Western blot and real-time RT-PCR in MRC-5 cells transfected with NC siRNA or Sirt2 siRNA for 24 h in the absence or presence of 2 ng/ml TGF-*β*1. **(E)** Densitometric analyses of the Western blot in **(D)**. **(G)** Expression of Sirt2, Fibronectin and *α*-SMA by Western blot in IPF lung fibroblasts treated with NC siRNA or Sirt2 siRNA for 24 h. **(H)** Densitometric analyses of the Western blot in **(G)**. GAPDH or *β*-actin was used as loading control. The bars indicated mean ± SD of three separate experiments. NC, negative control; siRNA, small interfering RNA. **p <0.01, ***p <0.001, ****p <0.0001.

To further examine the role of Sirt2 in lung fibroblasts activation, we used selective Sirt2 inhibitor AGK2 to inhibit its function in MRC-5 cells, and then analyzed the expression of Fibronectin and *α*-SMA. MRC-5 cells were treated with 10 μM AGK2 or vehicle control for 24 h in the presence of 2 ng/ml TGF-*β*1. The results showed that AGK2 significantly downregulated the increased protein and mRNA expression of Fibronectin and *α*-SMA induced by TGF-*β*1 (Figures 2A–C). Likewise, immunofluorescence staining further demonstrated that AGK2 treatment reversed the increased Fibronectin and *α*-SMA fluorescence intensity induced by TGF-*β*1 (Figure 3D). These data demonstrated that inhibiting Sirt2 can downregulate expression of Fibronectin and *α*-SMA at transcriptional and translational levels in TGF-*β*1-stimulated lung fibroblasts.

### Sirt2 siRNA Attenuates Fibrogenic Gene Expression in TGF-*β*1-Treated Lung Fibroblasts and IPF Lung Fibroblasts

After analyzing the function of Sirt2 by the pharmacologic inhibitor AGK2, two kinds of siRNA targeting Sirt2 were used to further confirm the role of Sirt2 in fibroblast activation. First, the high knockdown efficiency of two Sirt2 siRNAs was verified by Western blot and real-time RT-PCR (Figures 3A–C). Considering these two Sirt2 siRNAs have the same silent efficiency in down-regulating Sirt2 protein expression, so we only used Sirt2 siRNA1 in the following Sirt2 knockdown experiments. Sirt2 siRNA or NC siRNA transfected MRC-5 cells were incubated with TGF-*β*1 for 24 h, and the results showed that silencing Sirt2 expression significantly attenuated the protein (Figures 3D,E) and mRNA expression (Figure 3F) of Fibronectin and *α*-SMA induced by TGF-*β*1 stimulation. Similarly, in primary lung fibroblasts derived from IPF patients, silencing Sirt2 expression with siRNA decreased the expression levels of Fibronectin and *α*-SMA protein (Figures 3G,H). Overall, these data suggest that interfering the expression of Sirt2 suppresses lung fibroblasts activation.

### Inhibition of Sirt2 Alleviates the Increased Smad2/3 Phosphorylation Induced by TGF-*β*1

**FIGURE 4:**
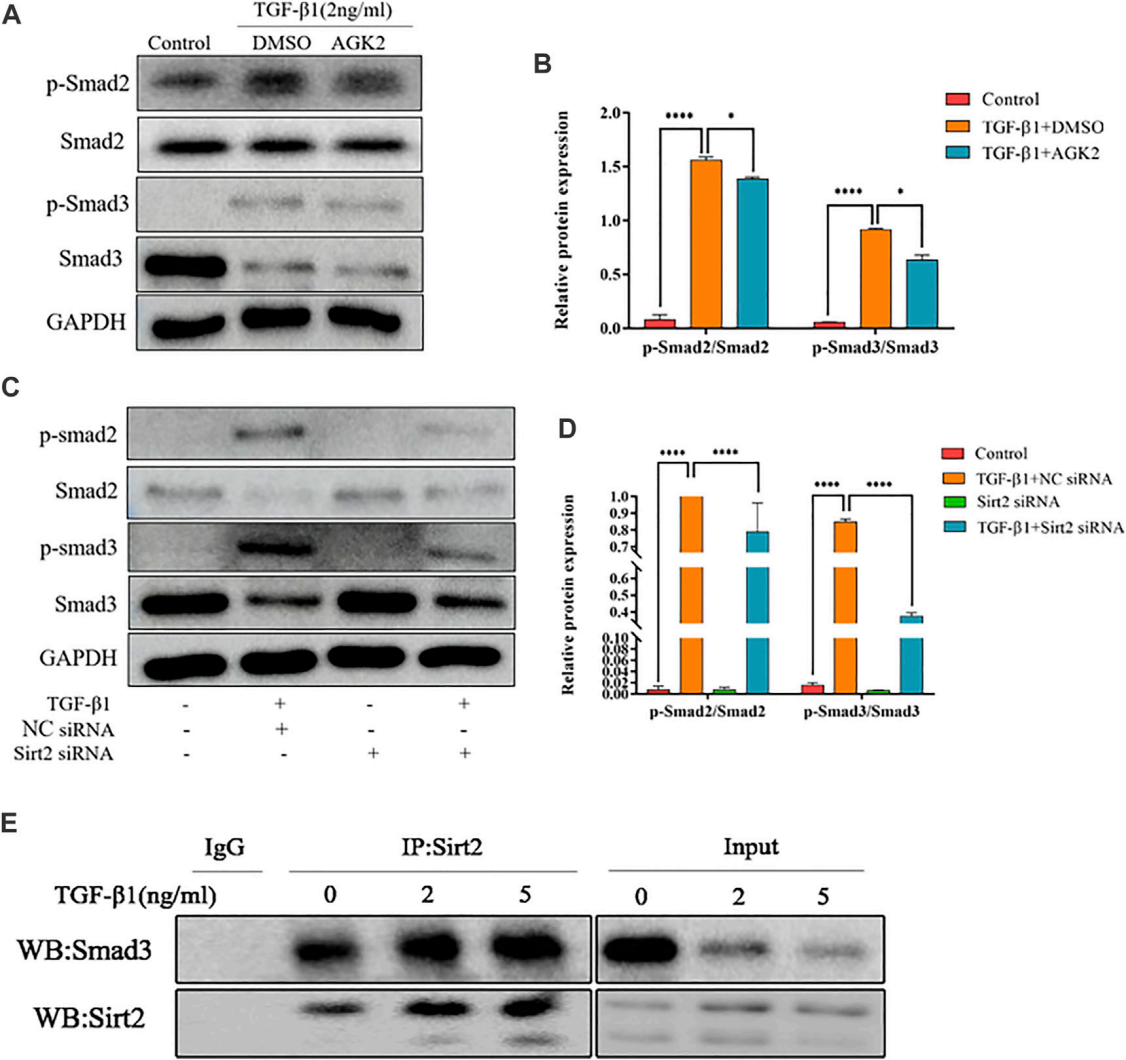
Inhibiting Sirt2 activity and expression downregulates the increased Smad2/3 phosphorylation induced by TGF-*β*1. **(A)** Protein **e**xpression of p-Smad2/Smad2 and p-Smad3/Smad3 by Western blot in MRC-5 cells pretreated with 2 ng/ml TGF-*β*1 for 24 h and then 10 μM AGK2 or DMSO for 24 h in the presence of TGF-*β*1. **(B)** Densitometric analyses of the Western blot in **(A)**. **(C)** Protein expression of p-Smad2/Smad2 and p-Smad3/Smad3 by Western blot in MRC-5 cells transfected with NC siRNA or Sirt2 siRNA for 24 h in the absence or presence of 2 ng/ml TGF-*β*1. **(D)** Densitometric analyses of the Western blot in **(C)**. **(E)** MRC-5 cells were treated with TGF-*β*1 at 2 and 5 ng/ml for 24 h, and total protein was co-immunoprecipitated with anti-Sirt2 antibody or IgG and immunoblotted with Smad3 antibody. GAPDH was used as a loading control. The bars indicated mean ± SD of three separate experiments. NC, negative control; siRNA, small interfering RNA; DMSO, dimethyl sulfoxide. *p <0.05, ***p <0.001.

TGF-*β*1/Smad2/3 is a well-known signaling pathway involved in tissue fibrosis. Upon TGF-*β*1 stimulation, Smad2/3 are phosphorylated and the phosphorylated Smad2/3 combines with Smad4 to form heteromeric complexes, which translocate into the nucleus to modulate target gene transcription (Yan et al., 2016). Several studies showed that some Sirtuins, including Sirt1, Sirt3, Sirt6, and Sirt7, involved in the pathogenesis of fibrosis partially through TGF-*β*1/Smad2/3 signaling pathway (Sosulski et al., 2017; Wyman et al., 2017; Zhang et al., 2019). Therefore, we hypothesized that Sirt2 also modulates TGF-*β*1 induced lung fibroblasts activation through Smad2/3 pathway. Our results showed that phospho-Smad2 (p-Smad2) and phospho-Smad3 (p-Smad3) levels were significantly upregulated in response to TGF-*β*1 treatment compared with control (Figures 4A–D). AGK2 or Sirt2 siRNA treatment downregulated the increased phosphorylation of Smad2/3 induced by TGF-*β*1, decreased total Smad3 protein was also observed when Sirt2 was inhibited (Figures 4A–D). These indicated that Sirt2 promote the lung fibroblasts activation in a Smad2/3-dependent manner. Co-IP demonstrated directly that Sirt2 interacts with Smads in TGF-*β*1 treated MRC-5 (Figure 4E). Taken together, these results suggest that Sirt2 regulates fibroblasts activation through Smad2/3 signaling pathway in human embryonic lung fibroblasts.

### AGK2 Alleviates Bleomycin-Induced Pulmonary Fibrosis in Mice

**FIGURE 5:**
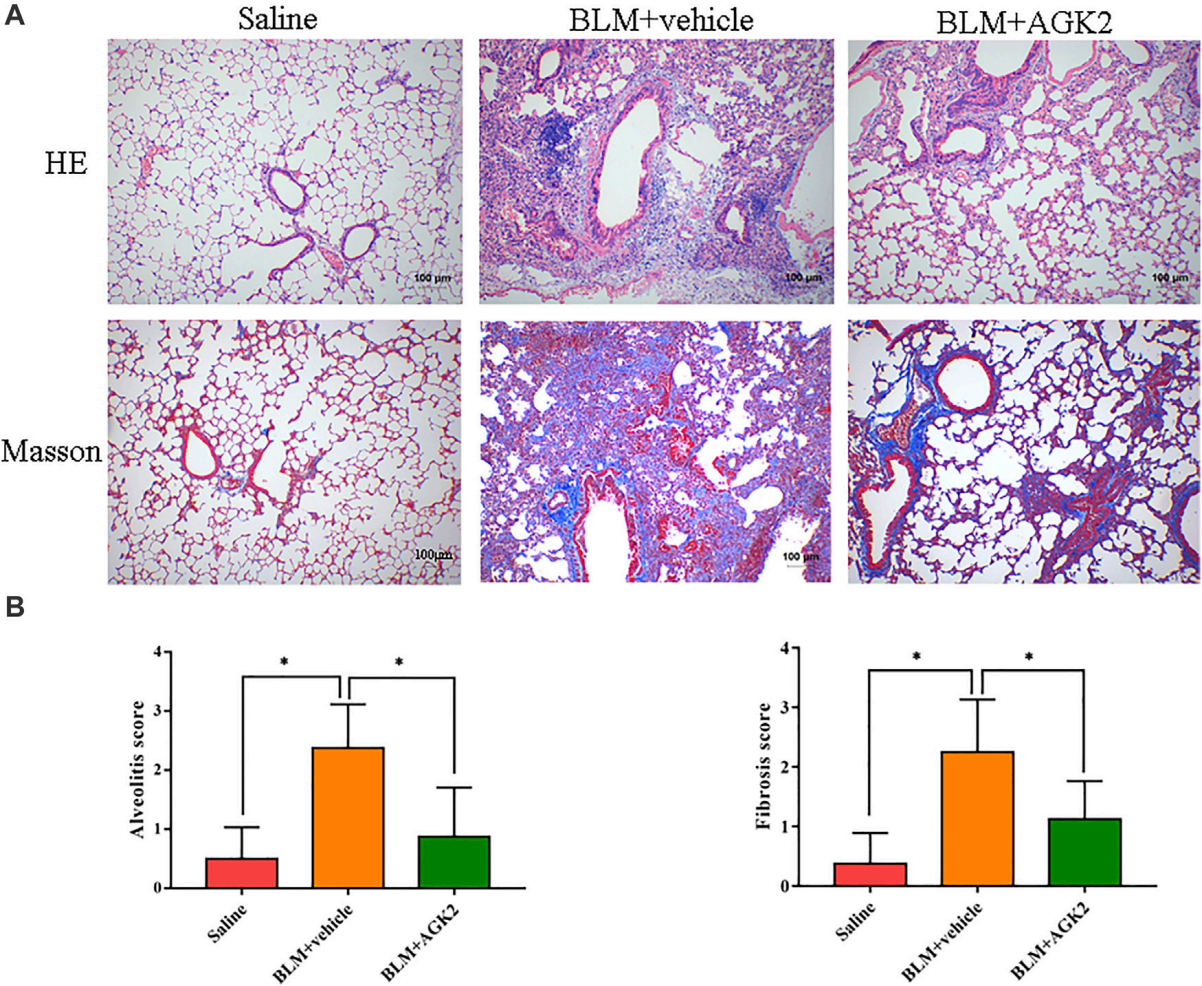
AGK2 alleviates the degree of pulmonary fibrosis in bleomycin-induced pulmonary fibrosis in mice. **(A)** Representative images of HE and Masson staining of lung tissues. Magnification, ×100. **(B)** The quantitative results of alveolitis and fibrosis scoring. BLM, bleomycin. *p <0.05.

Mice model of bleomycin-induced pulmonary fibrosis was used to elucidate the protective effects of AGK2 treatment *in vivo*. Lung tissues were examined with HE and Masson staining. In HE staining, saline-treated lung tissue showed normal alveolar spaces and normal thickening of the alveolar septa; bleomycin stimulation induced obviously more interstitial infiltration by inflammatory cells than saline-treated control group, AGK2 administration apparently attenuated the degree of alveolitis (Figure 5A upper panel). In Masson staining, bleomycin stimulation induced a significant thickening of the alveolar septa with increased deposition of collagen in lung tissues compared with the control (Figure 5A lower panel). Quantitative analysis showed that the alveolitis and fibrosis scores induced by bleomycin were significantly reduced after AGK2 treatment (Figure 5B).

### AGK2 Alleviates Bleomycin-Induced Pulmonary Fibrosis and Decreases the Expression of p-Smad2/3 *in vivo*

**FIGURE 6:**
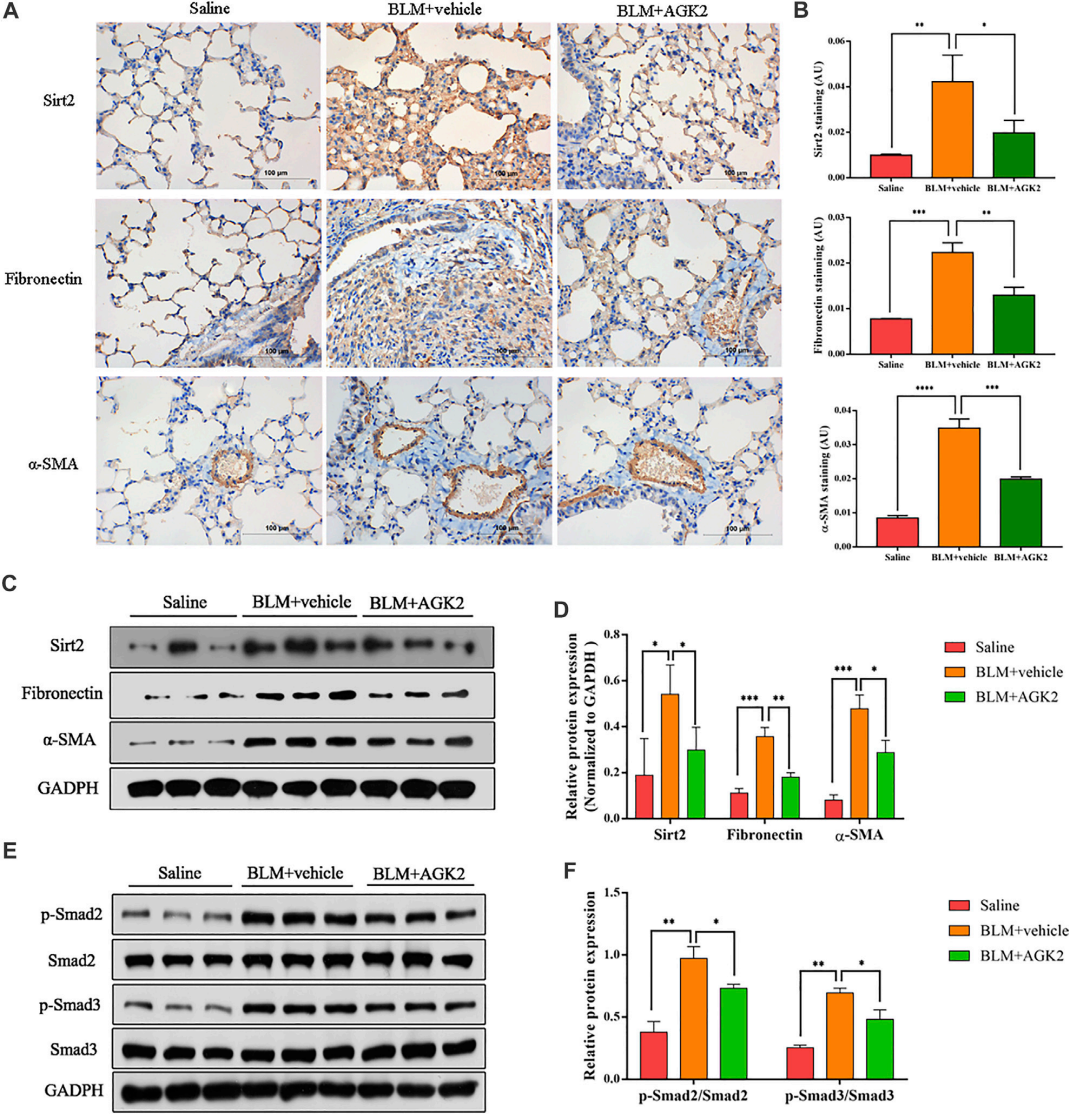
AGK2 attenuated bleomycin-induced pulmonary fibrosis and decreased the expreesion of p-Smad2/3 *in vivo*. **(A)** Representative image of IHC staining of Sirt2 (top, brown), Fibronectin (middle, brown) and *α*-SMA (bottom, brown) (magnification: ×400). **(B)** Quantitative analysis of IHC in **(A)** with Image-Pro Plus 6.0 software. **(C,E)** Protein expression of Sirt2, Fibronectin, *α*-SMA, p-Smad2/Smad2 and p-Smad3/Smad3 of lung tissues by Western blot. **(D,F)** Densitometric analyses of the Western blot in **(C,E)**. BLM, bleomycin. *p <0.05, **p <0.01, ***p <0.001, ****p <0.0001.

In mice model of bleomycin-induced pulmonary fibrosis, IHC was performed to further explore the effects of AGK2 on the expression of fibrosis-related proteins. As shown in Figures 6A,B, the staining of Fibronectin, *α*-SMA, and Sirt2 protein in the saline group was not remarkable, while the positive staining showed as dark brown was significantly increased after treating with bleomycin. Western blot showed similar results, which demonstrated the protein expression of Sirt2, Fibronectin and *α*-SMA were higher in the lung tissue of bleomycin-treated mice, but lower in those with AGK2 treatment, when compared to the saline control (Figures 6C,D). Furthermore, AGK2 treatment significantly decreased the levels of p-Smad2/Smad3 induced by bleomycin (Figures 6E,F). These results demonstrated that Sirt2 inhibitor can alleviated bleomycin-induced pulmonary fibrosis *in vivo* and inactivated Smad2/3 signaling pathway.

## Discussion

IPF is a progressively fatal disease and more effective therapeutic strategies are urgently needed. However, the underlying mechanism of the progress of IPF has not yet been fully elucidated. The chronic injuries or repetitive stimulation of lung epithelial cells lead to aberrantly activated fibroblast proliferation and excessive amount of ECM deposition may be a key process for this disease. Emerging evidence suggests that other Sirtuins are involved in the fibroblast activation and progression of pulmonary fibrosis (Mazumder et al., 2020). For the first time, we demonstrate that the expression of Sirt2 is increased in lung fibroblasts stimulated with TGF-*β*1 *in vitro* and in the mice model of pulmonary fibrosis induced by bleomycin *in vivo*; inhibiting Sirt2 by the pharmacologic inhibitor or targeting small interfering RNA can inhibit fibrosis process by blocking Smad2/3 signaling pathway.

The occurrence and development of pulmonary fibrosis is complex. TGF-*β*1 is the most important primary driver and mediator in the process of pulmonary fibrosis through recruiting and activating fibroblasts, promoting epithelial-mesenchymal transition (EMT) and inducing ECM production (Hu et al., 2018). TGF-*β*1 regulates a complex networks of gene expression, including Smad and Sirtuins signaling pathway.

The mechanisms by which the Sirtuins contribute to the pathogenesis of fibrotic diseases are different in previous studies. Among these Sirtuins, Sirt1, Sirt3, Sirt6, and Sirt7 have been well studied in pulmonary fibrosis (Chun, 2015; Mazumder et al., 2020). Several studies have documented the regulatory function of Sirtuins on some classic fibrotic-related signaling pathway including TGF-*β*1/Smads. For instance, Sirt1 activation or overexpression can inhibit pulmonary fibrosis *in vitro* via inactivation of TGF-*β*1/Smad3 and mTOR signaling (Warburton et al., 2013; Chu et al., 2018). Sirt6 inhibits lung myofibroblasts differentiation by repressing NF‐κB‐dependent transcriptional activity and TGF-*β*1/Smad2 signaling pathway (Tian et al., 2017; Zhang et al., 2019).

Previous studies have demonstrated that Sirt2 is involved in the pathological process of tissue fibrosis. A pro-fibrotic function of Sirt2 has been documented in hepatic fibrosis. Genetic or pharmacological inhibition of Sirt2 significantly suppressed fibrogenic gene expression in hepatic stellate cells through ERK dephosphorylation and c-MYC degradation (Arteaga et al., 2016). In the study of hepatitis B virus (HBV) infection, Sirt2 overexpression was associated with Akt activation, which consequently downregulated glycogen synthase kinase 3*β* (GSK-3*β*) and increased *β*-catenin levels. These results indicate that Sirt2 inhibitor may control HBV infection and prevent the development of hepatic fibrosis (Piracha et al., 2018). In kidney fibrosis, AGK2 dose- and time-dependently inhibited the expression of fibrotic markers (Ponnusamy et al., 2014). Moreover, a stimulatory function of Sirt2 on eosinophil recruitment and inflammatory cytokines (TNF-*α*, IL-1*β*, IL-4 and IL-6) and mediators (myeloperoxidase, eosinophil peroxidase, and tumor growth factor-*α*) secretion in lung tissues was observed (Lee et al., 2019; Kim et al., 2020). However, the role of Sirt2 has not been explored in pulmonary fibrosis.

In this study, we showed that Sirt2 level was upregulated in TGF-*β*1 activated human lung fibroblasts and lung tissues of bleomycin-treated mice model, which suggested that Sirt2 may play a role in fibroblasts activation and pulmonary fibrogenesis. Downregulation of Sirt2 expression using pharmacologic inhibitor AGK2 and siRNAs alleviated TGF-*β*1 induced lung fibroblasts activation, as evidenced by reduced expression of *α*-SMA and Fibronectin. The anti-fibrotic effect of Sirt2 knockdown was also observed in IPF lung fibroblasts. Moreover, AGK2 treatment significantly mitigated the degree of pulmonary fibrosis in mice induced by bleomycin.

Smad2 and Smad3 are key mediators of TGF-*β*1-induced fibrogenesis and ECM production. TGF-*β*1 binds to its receptor and forms complexes with Smad2/3, then the phosphorylated Smad2/3 and its subsequent complex translocate to the nucleus, which are the key steps to modulate TGF‐*β*1 dependent gene expression and fibrosis progress (Li et al., 2019; Zou et al., 2019; Nanri et al., 2020). Our results found that p-Smad2/3 expression was increased in activated lung fibroblasts induced by TGF-*β*1 and in lung tissues of bleomycin-induced pulmonary fibrosis. Sirt2 inhibitor AGK2 or Sirt2 siRNA can attenuated its expression. Co-IP further identify the interactions between Sirt2 and Smad3. Our results illustrated that Sirt2 possibly regulates Smad2/3 directly or indirectly and lead to higher phosphorylation of Smad2/3 in response to stimulators; Sirt2 may promote the activation of fibroblasts and the development of pulmonary fibrosis through Smad2/3 pathway. This result was similar to a previous study, which demonstrated that AGK2 reduced the level of collagen deposition in specific Smad signaling transfected cells (Kim et al., 2020).

In the present study, Sirt2 has been identified as an important factor in the process of pulmonary fibrosis, and inhibition of Sirt2 ameliorated the degree of fibrosis and decreased the phosphorylation of Smad2/3, which indicate that targeting Sirt2 would provide novel therapeutic candidate for preventing pulmonary fibrosis. However, the limitation of this study is that only small molecule inhibitor was used, and further studies with Sirt2-KO mice are needed to investigate the exact effects of Sirt2 on pulmonary fibrosis.

## Data Availability

The original contributions presented in the study are included in the article/Supplementary Material, further inquiries can be directed to the corresponding author.
