# Supplementary material for: Inhibition of Sirt2 Alleviates Fibroblasts Activation and Pulmonary Fibrosis via Smad2/3 Pathway
Source: Front Pharmacol. 2021 Dec 1;12:756131. doi: 10.3389/fphar.2021.756131 (PMC8672210; doi:10.3389/fphar.2021.756131)

Fibronectin(control)


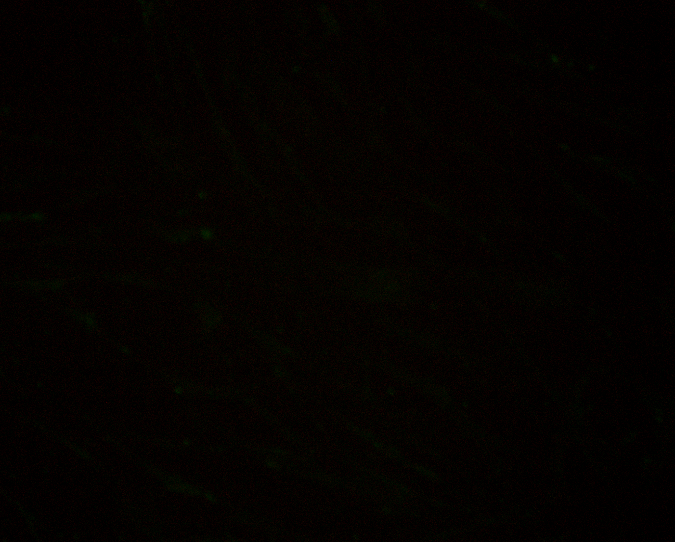

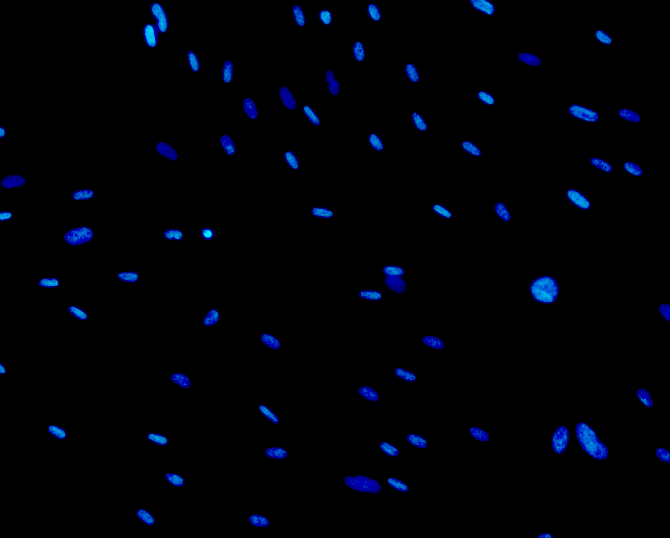


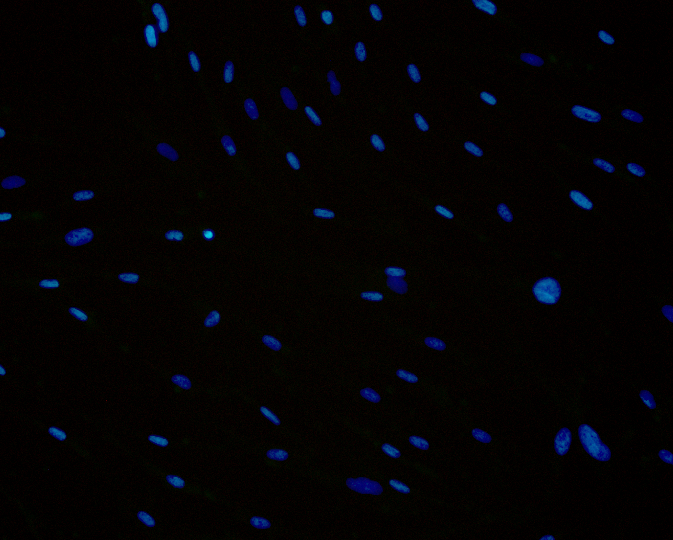


Fibronectin(TGF+DMSO)


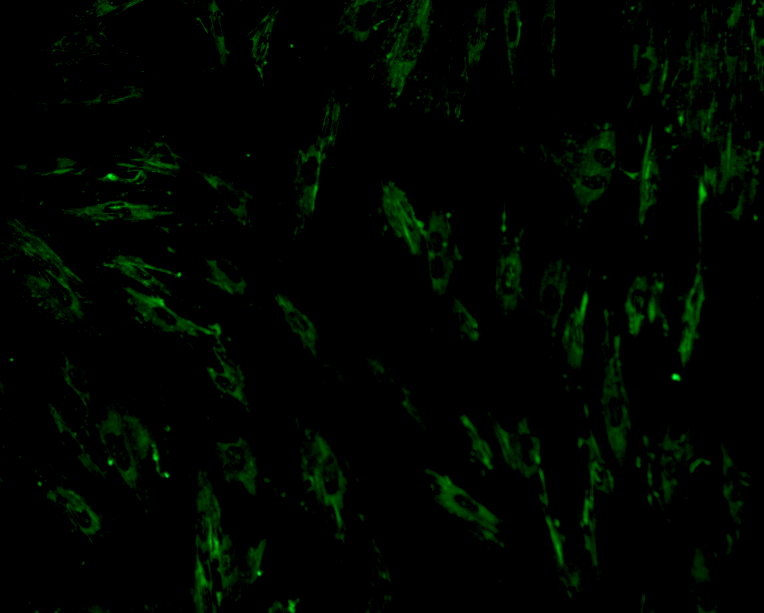


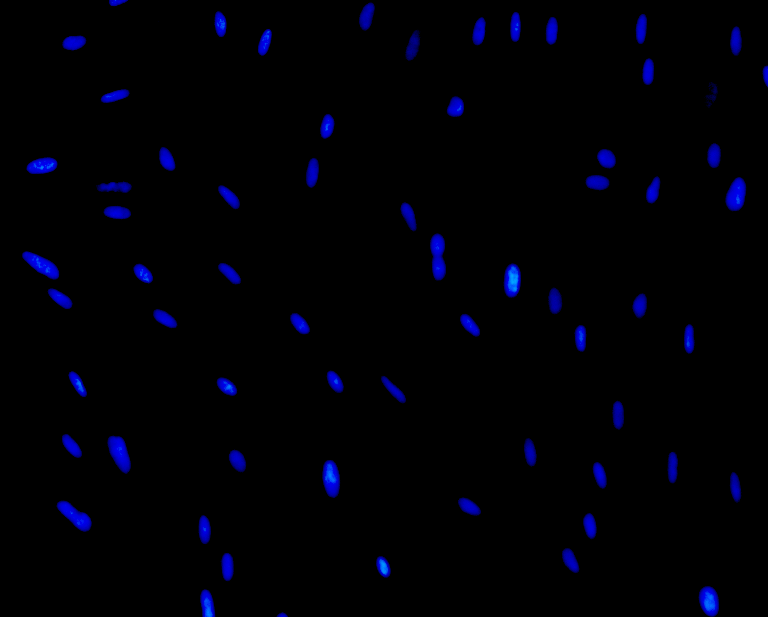


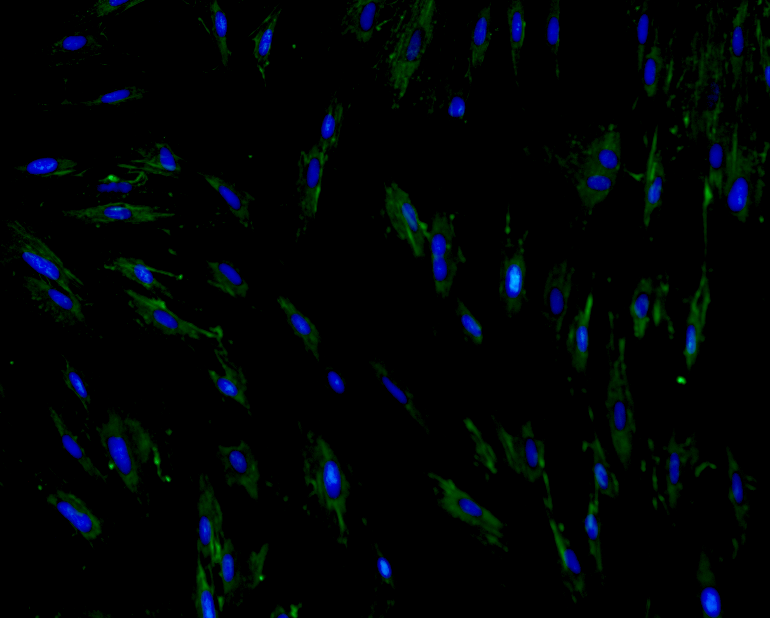


Fibronectin(TGF+AGK2)


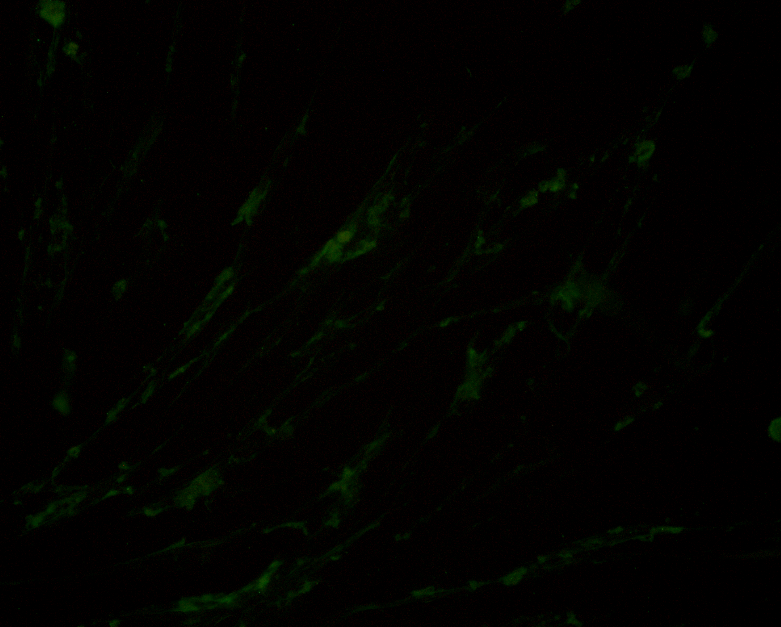


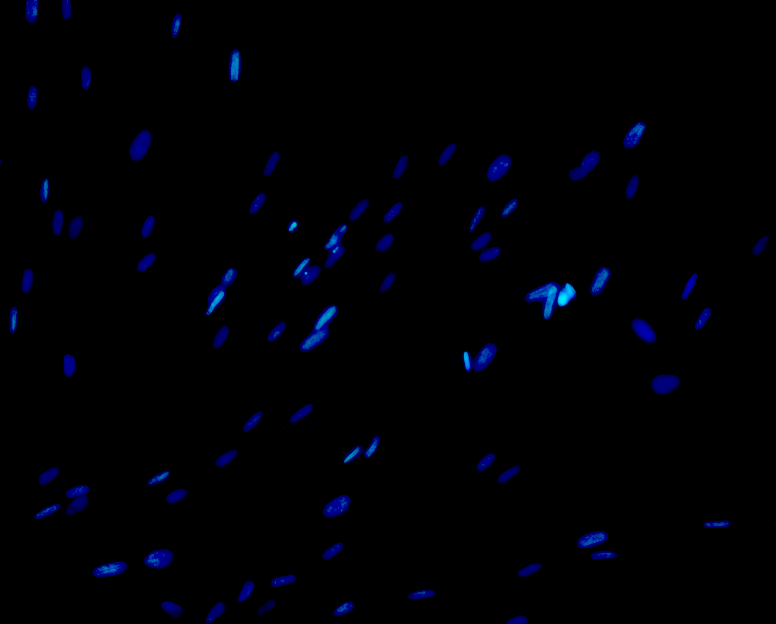


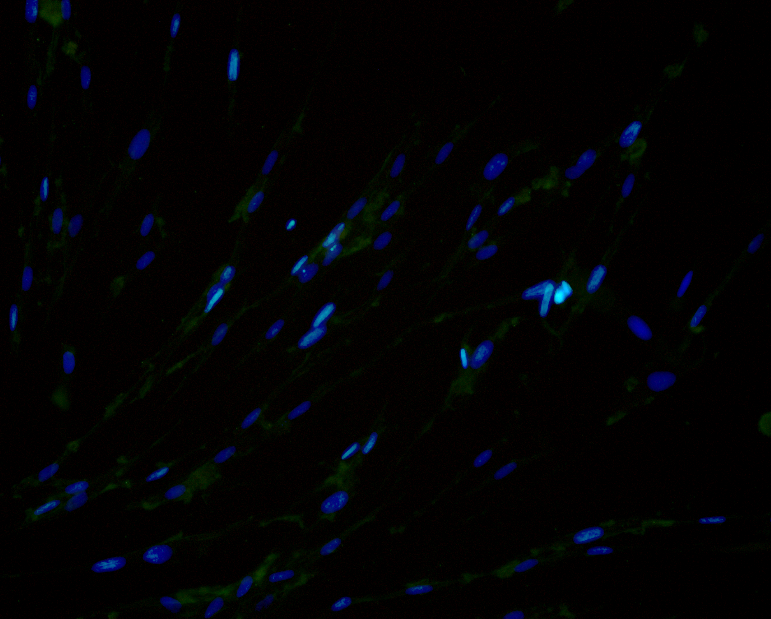


a-SMA(control)


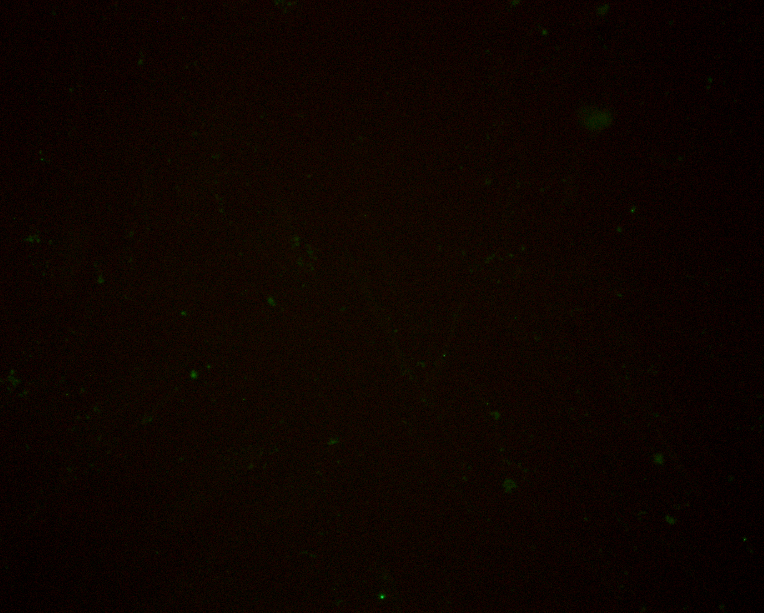


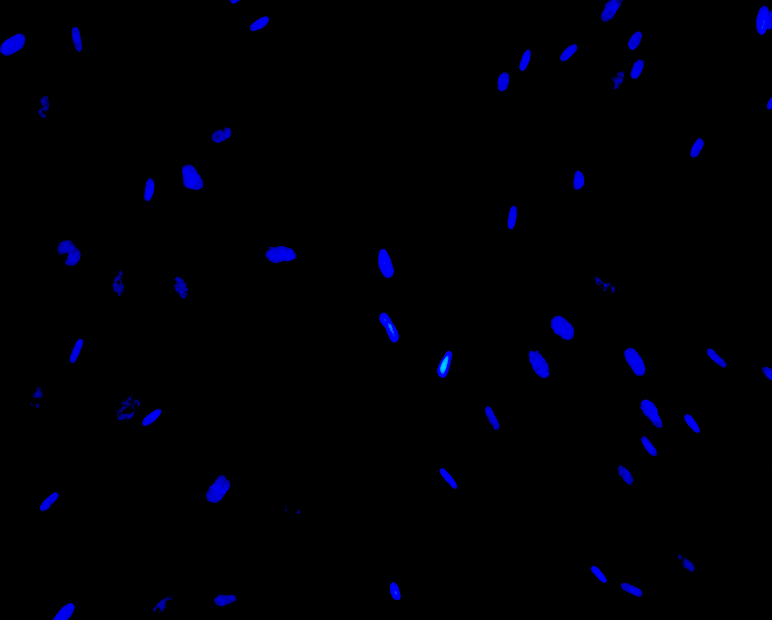


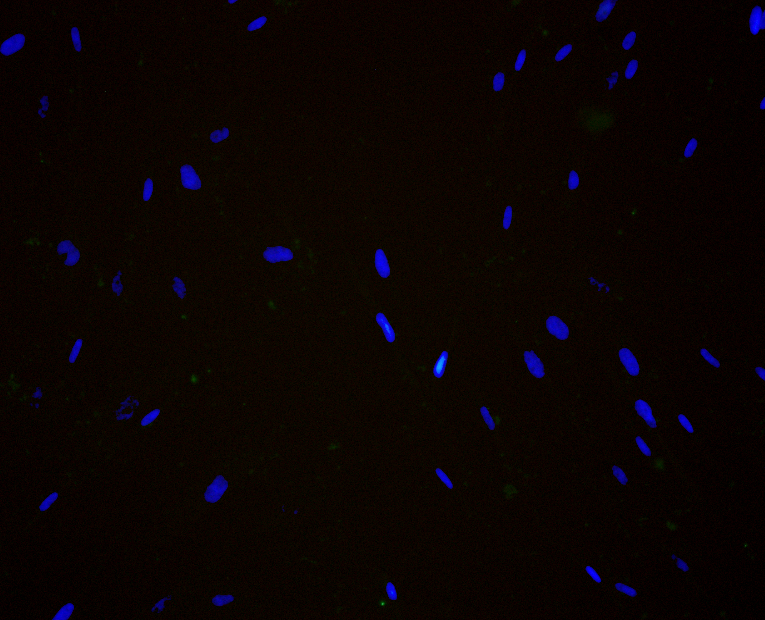


a-SMA (TGF+DMSO)


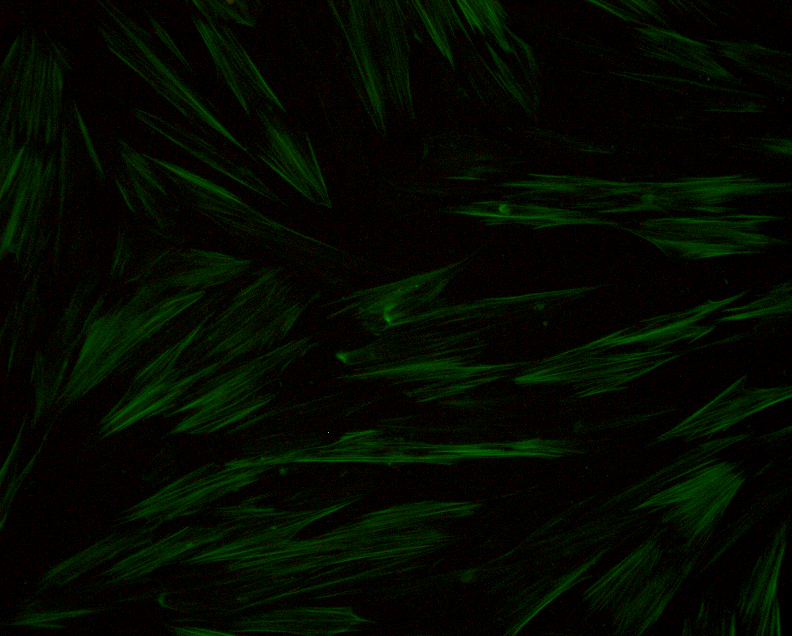


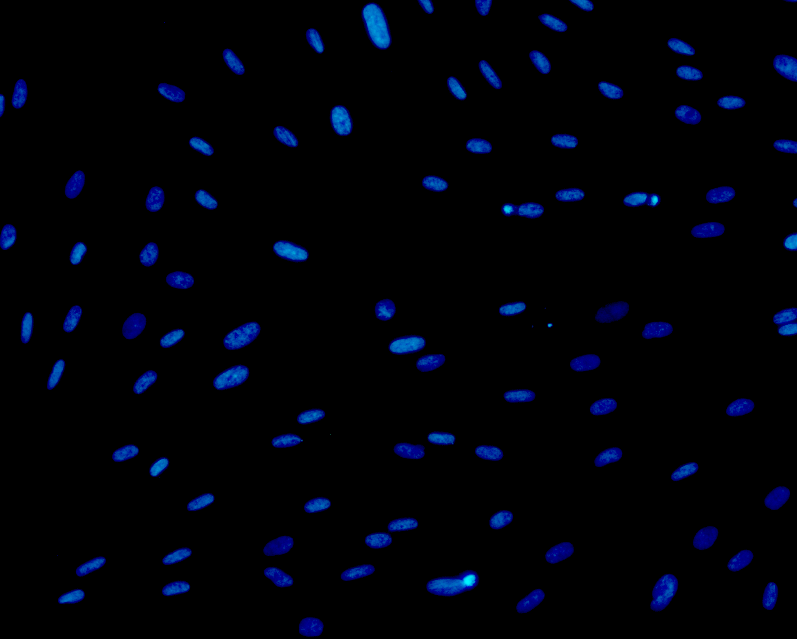


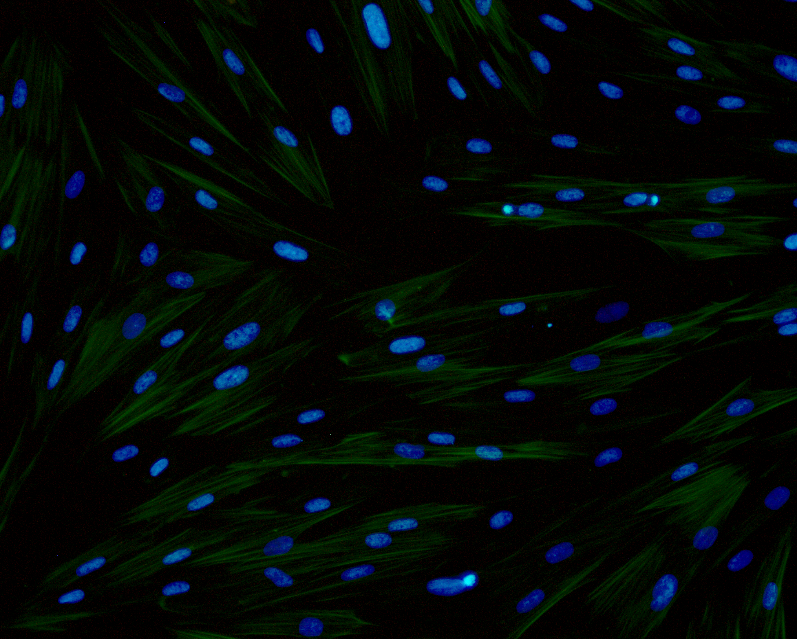


a-SMA (TGF+AGK2)


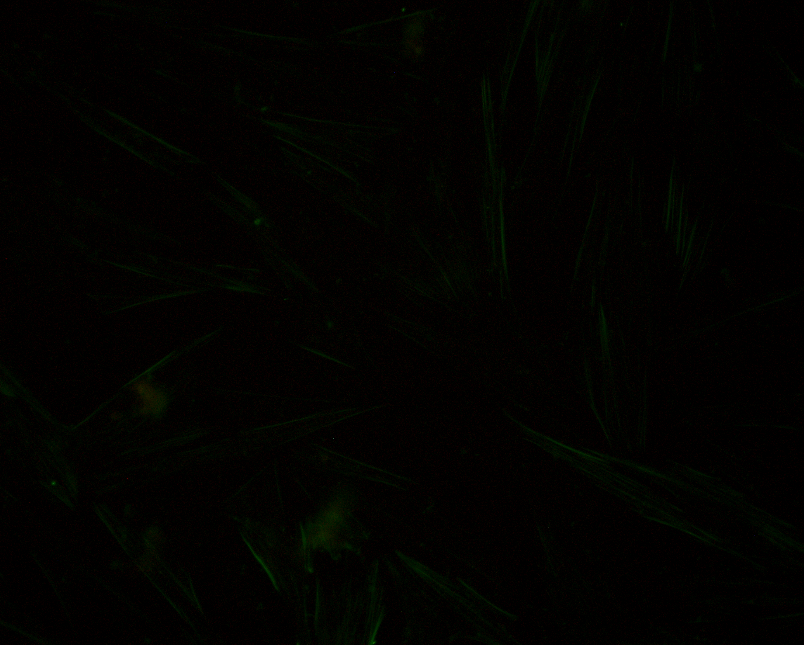


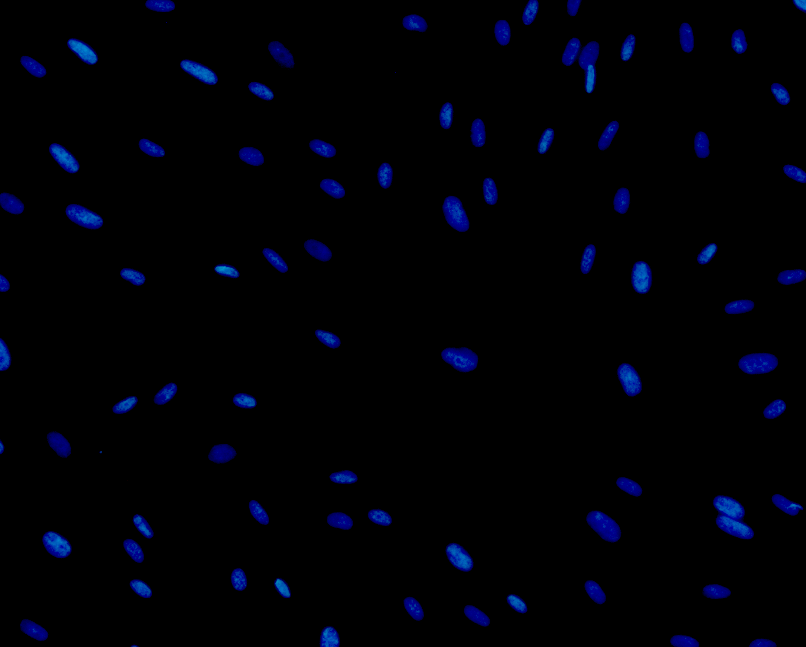


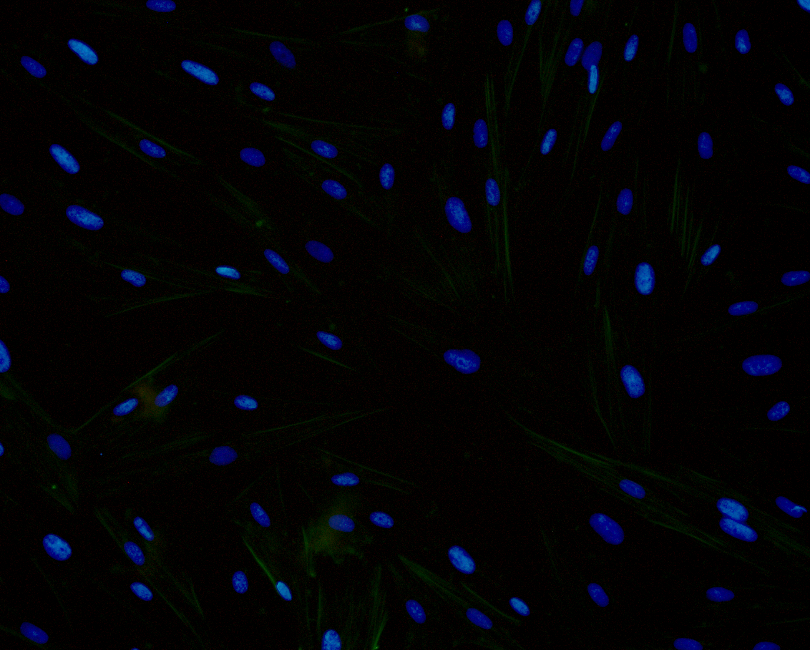

Supplement: Supplementary file 1 [file DataSheet1.zip › original data of sirt2/immunofluorescence staining.docx]
