# Supplementary material for: Inhibition of Sirt2 Alleviates Fibroblasts Activation and Pulmonary Fibrosis via Smad2/3 Pathway
Source: Front Pharmacol. 2021 Dec 1;12:756131. doi: 10.3389/fphar.2021.756131 (PMC8672210; doi:10.3389/fphar.2021.756131)
Supplement: Supplementary file 1 [file DataSheet1.zip › original data of sirt2/the data of animal study.docx]

Fig1. HE staining


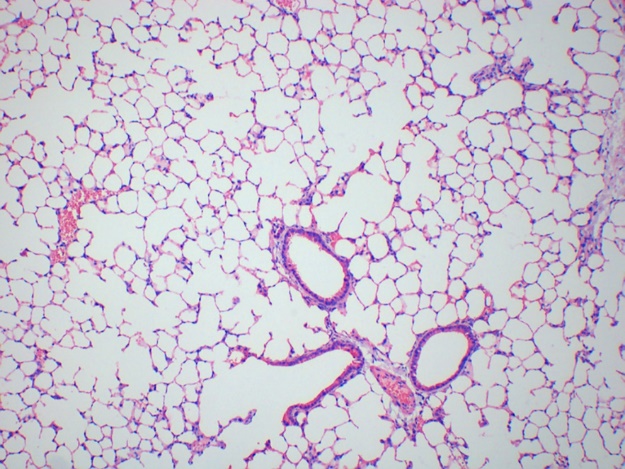

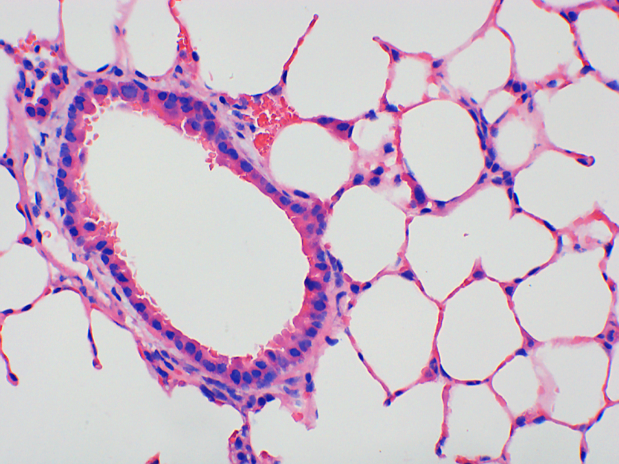


Control: left 100X, right 400X


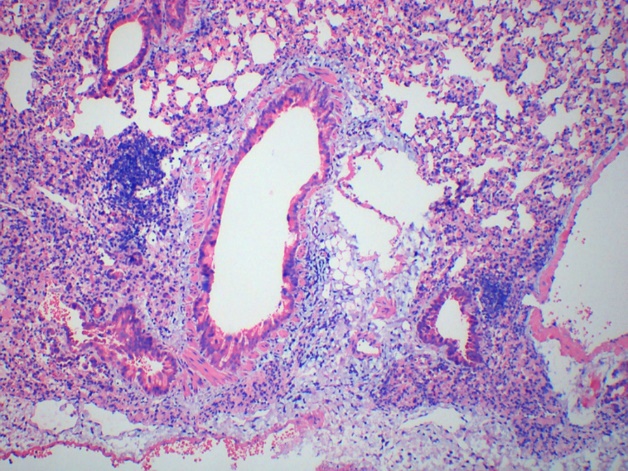

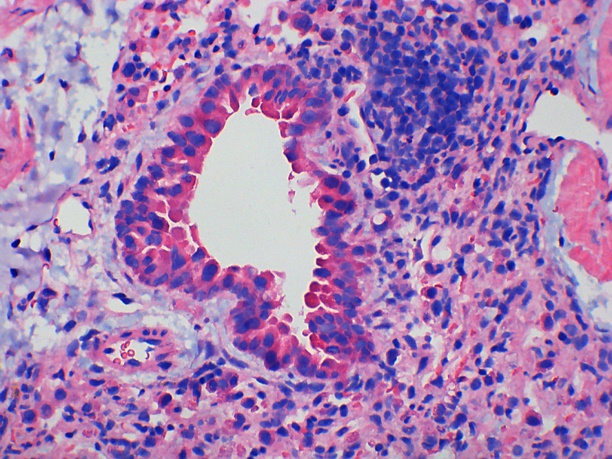


BLM+vehicle: left 100X, right 400X


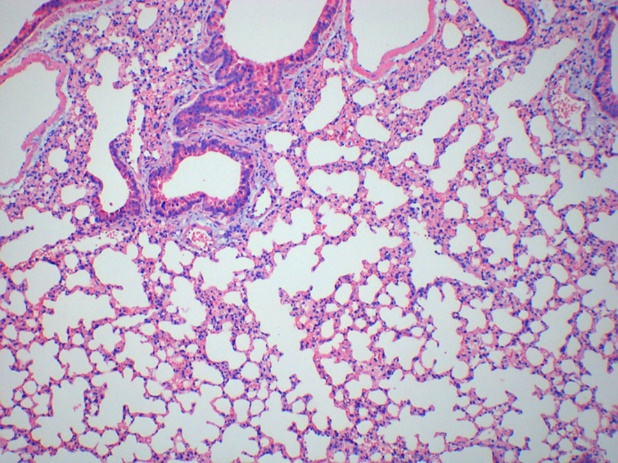

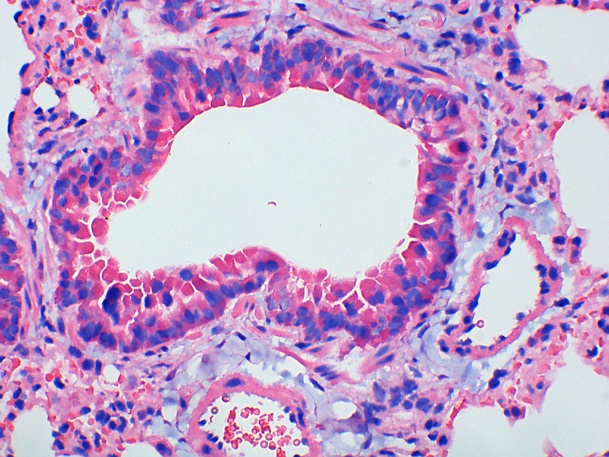


BLM+AGK2: left 100X, right 400X

Fig2. Masson staining


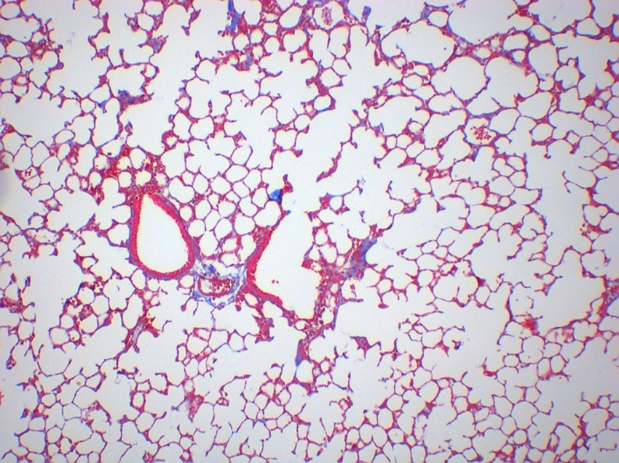

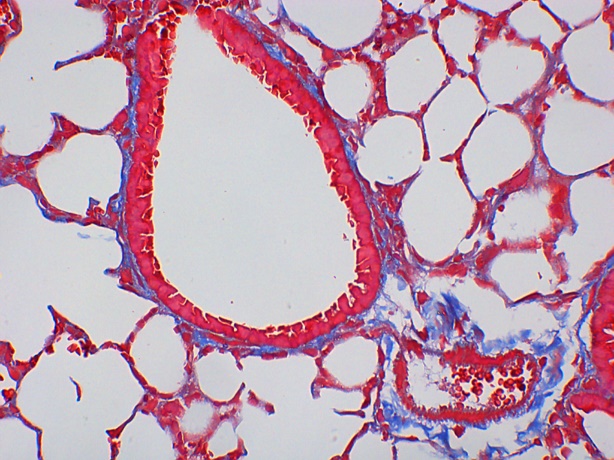


control: left 100X, right 400X


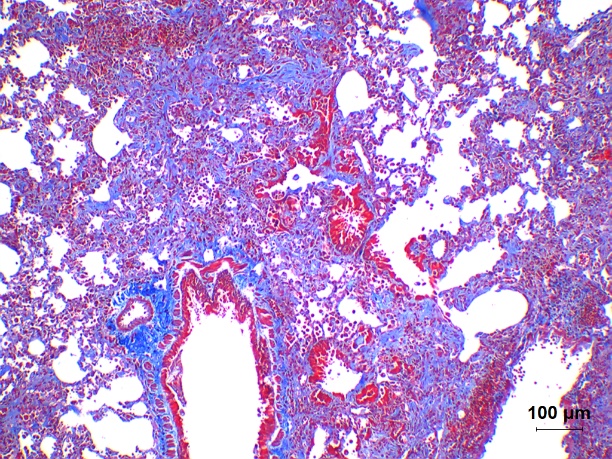

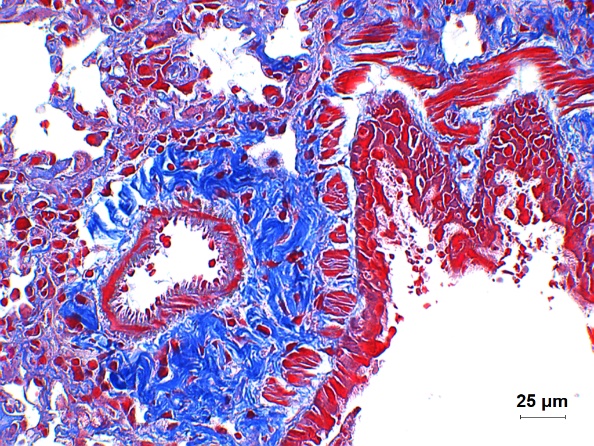


BLM+vehicle: left 100X, right 400X


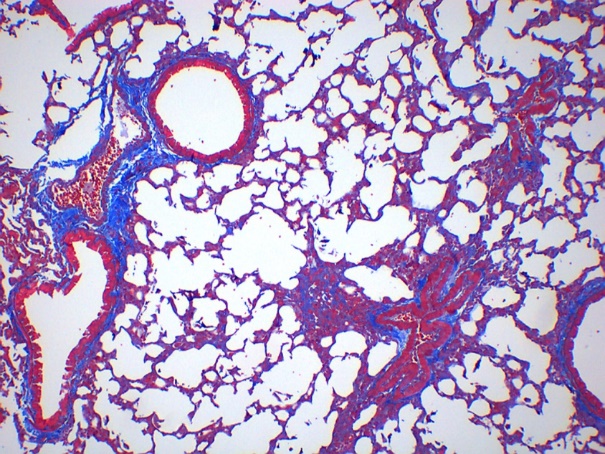

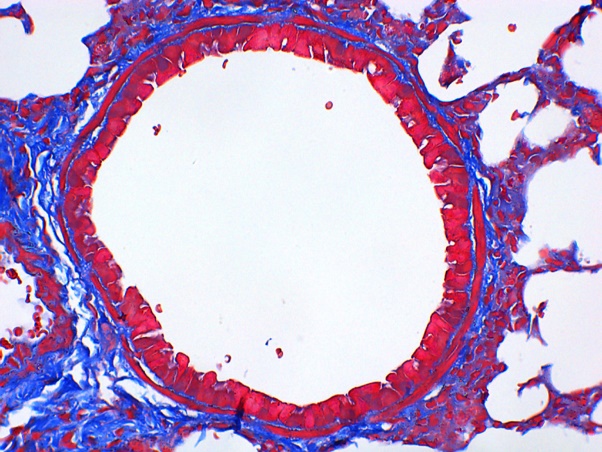


BLM+AGK2: left 100X, right 400X

Fig3A. Immunohistochemical staining of Sirt2


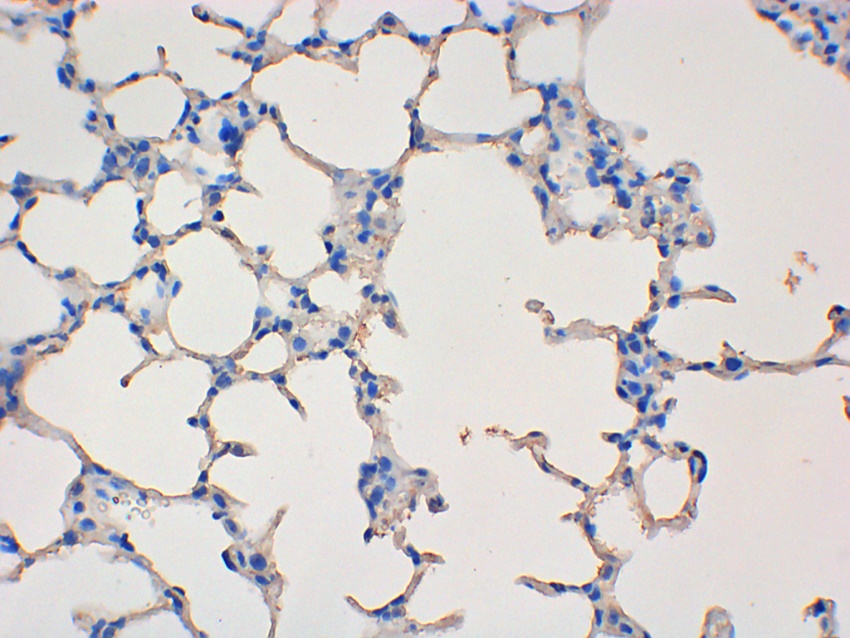
Sirt2(control)


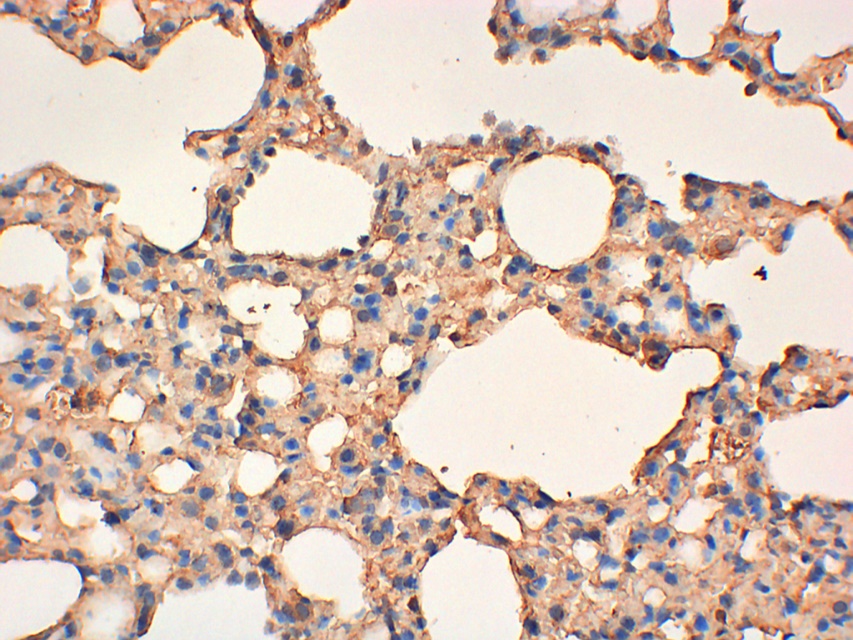
Sirt2(BLM+vehicle)


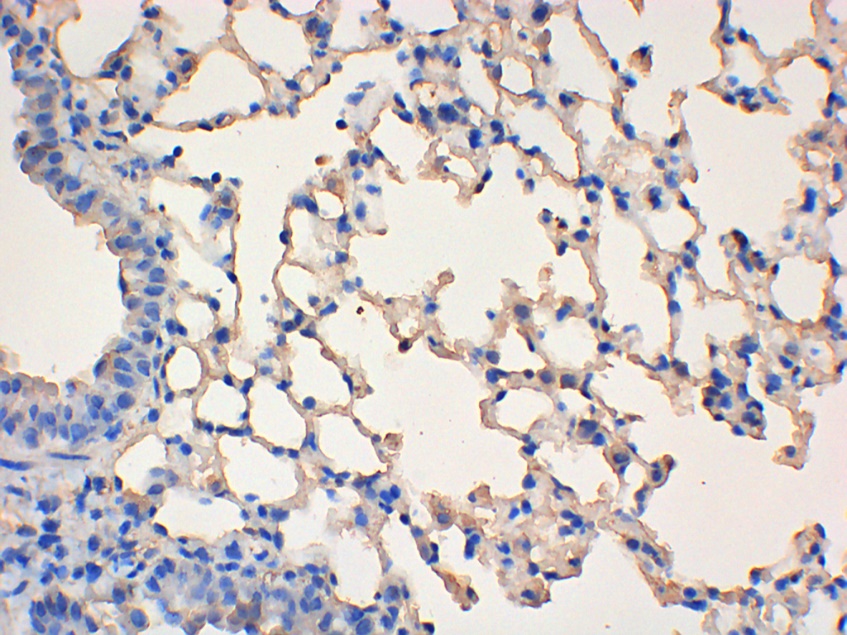
Sirt2(BLM+AGK2)

Fig3B. Immunohistochemical staining of fibronectin


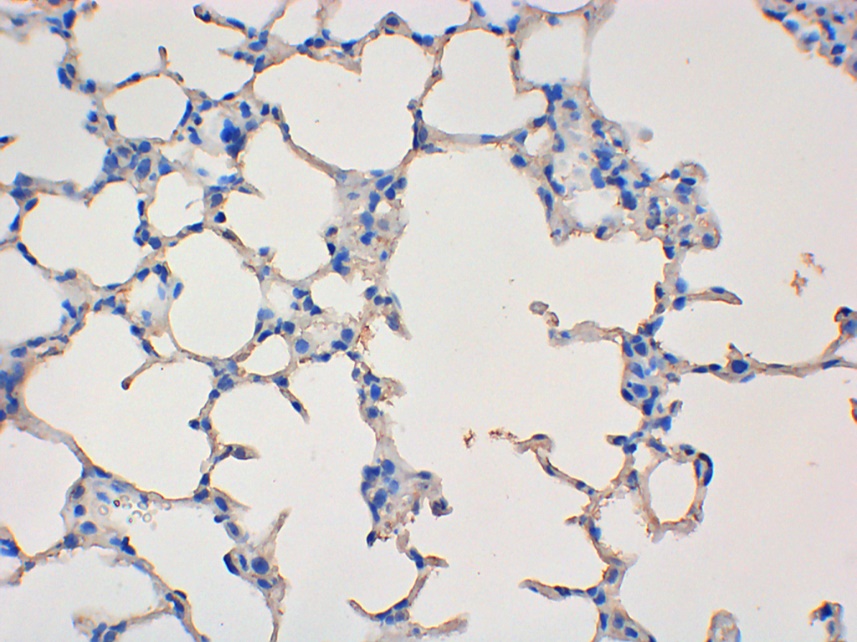
Fibronectin (control)


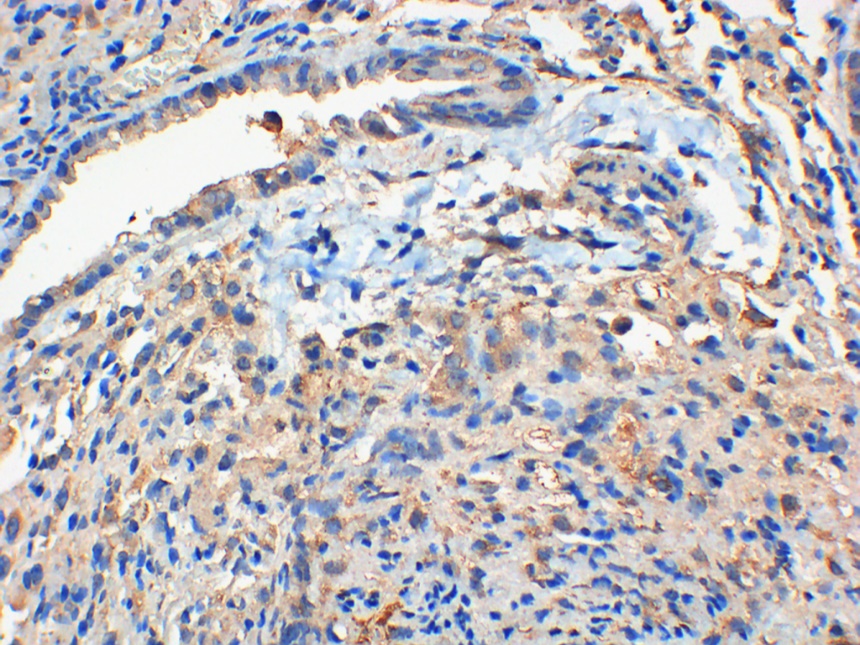
Fibronectin (BLM+vehicle)


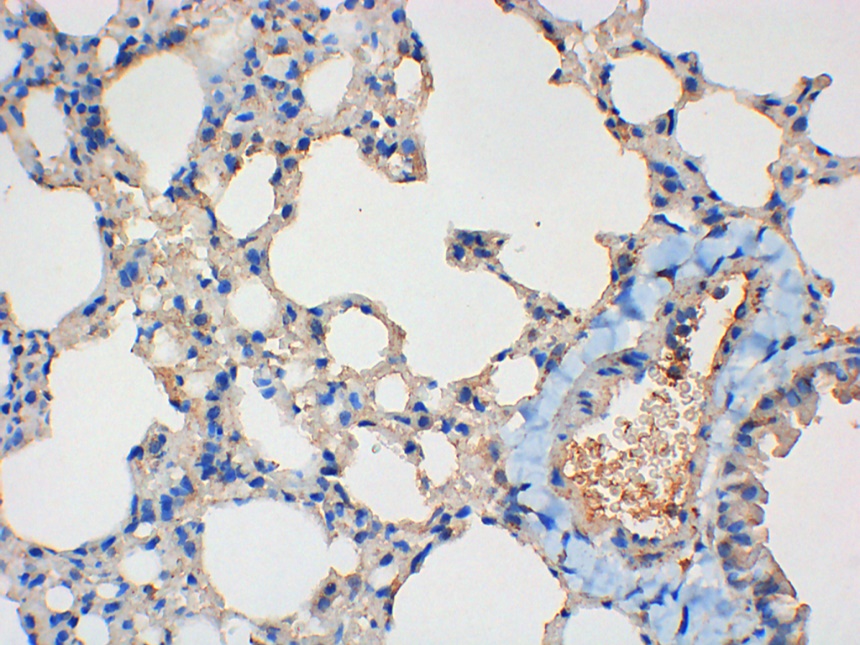
Fibronectin (BLM+AGK2)

Fig3C. Immunohistochemical staining of α-SMA


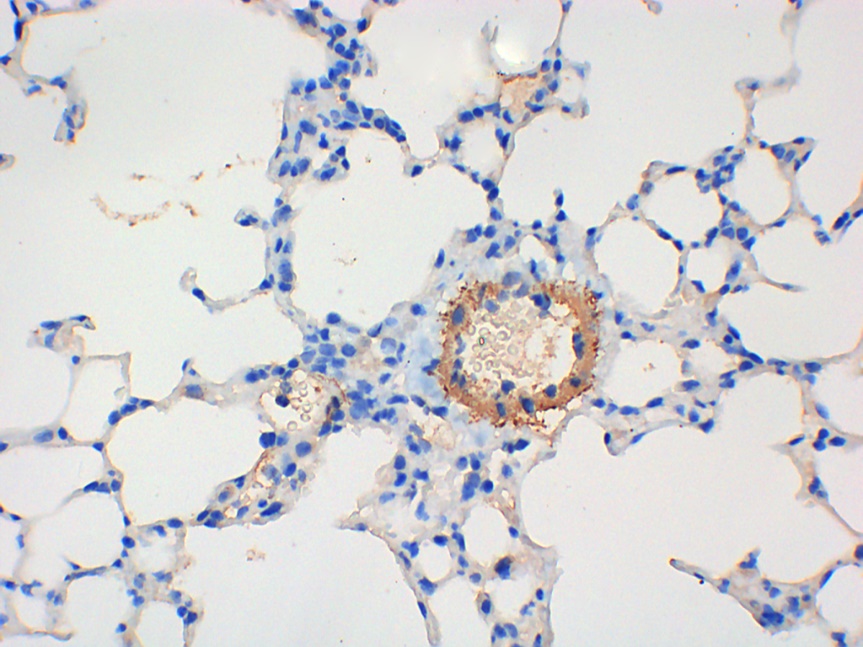
α-SMA (control)


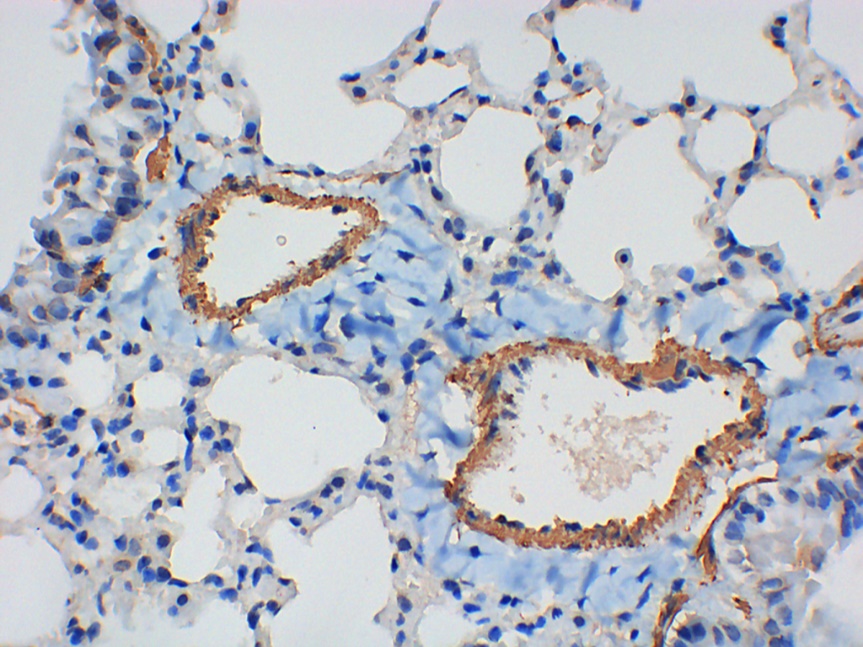
α-SMA (BLM+vehicle)


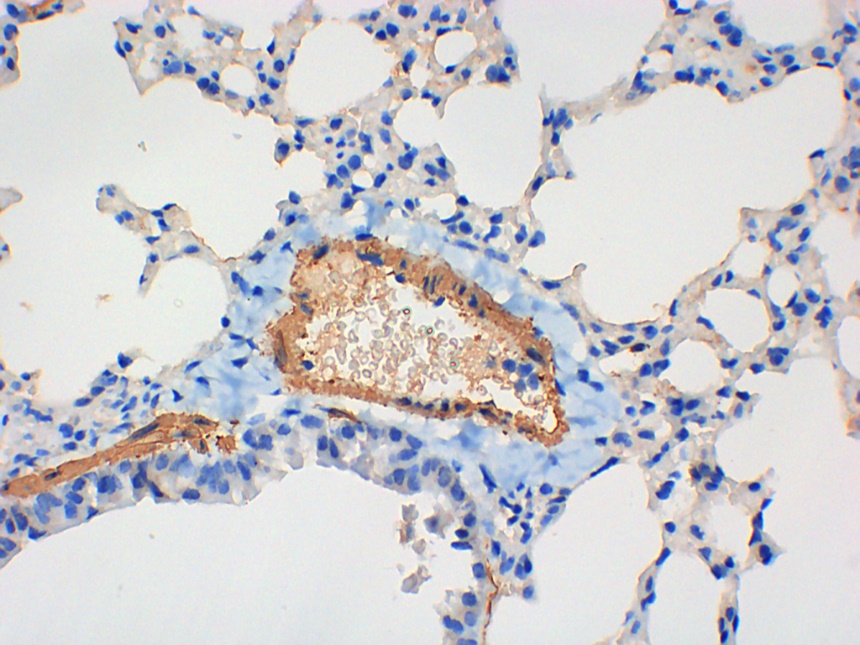
α-SMA (BLM+AGK2)

Analysis data of IHC


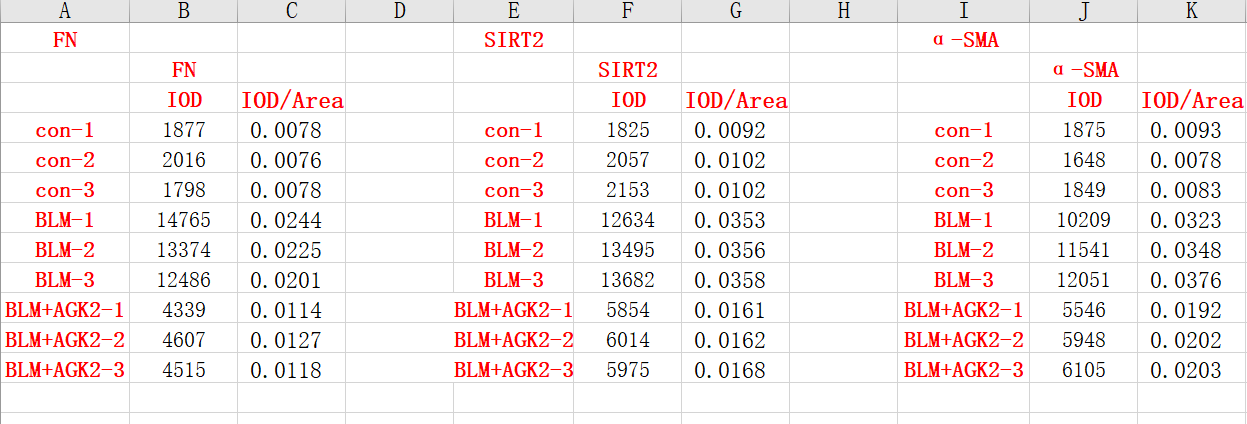


Western blot


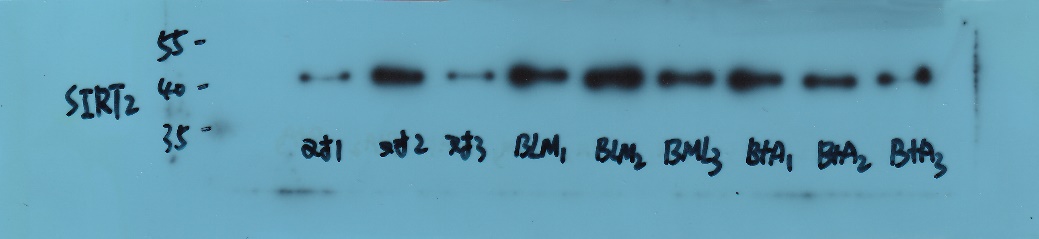


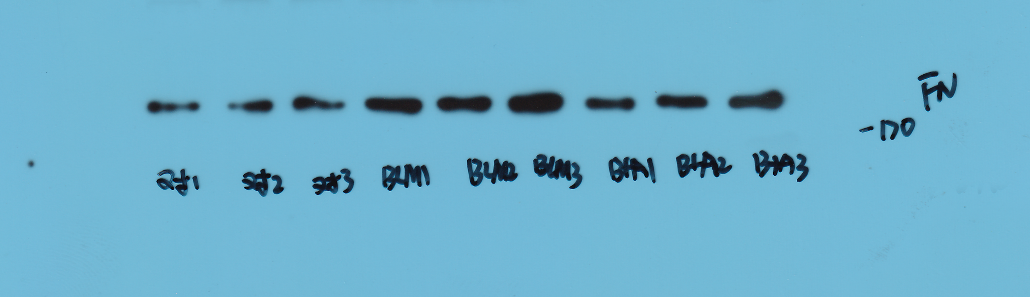


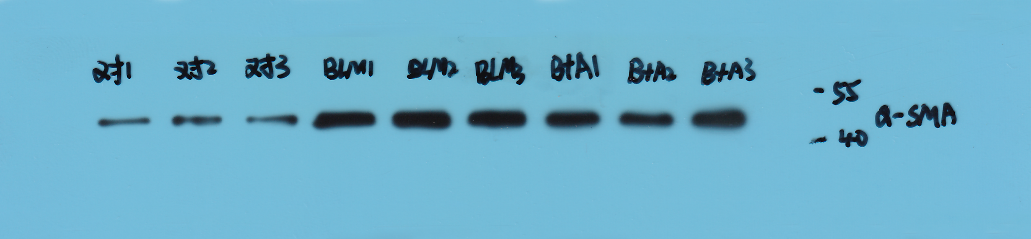


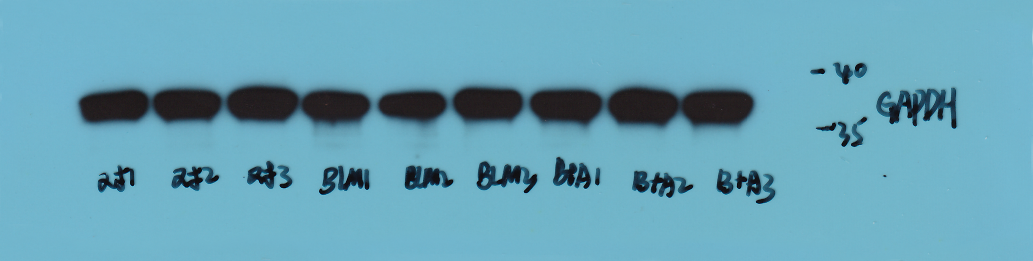


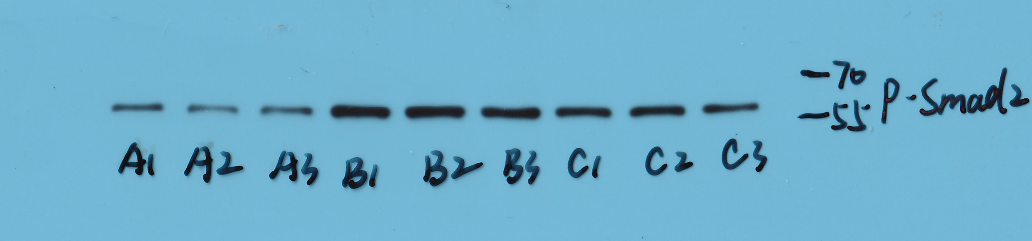


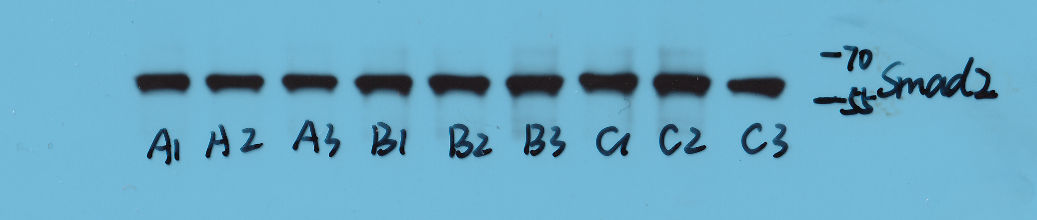


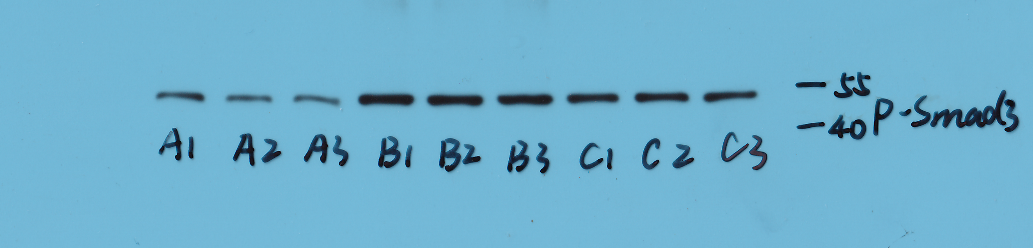


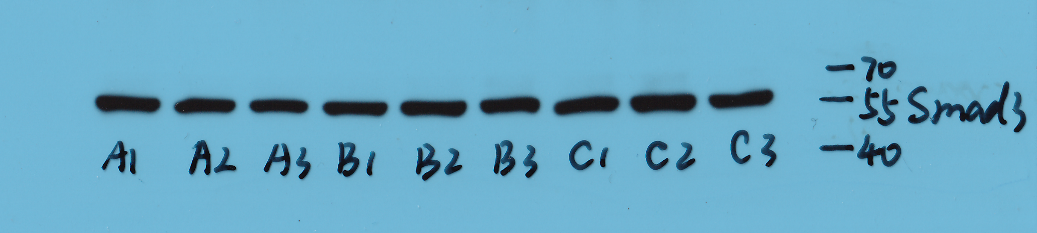


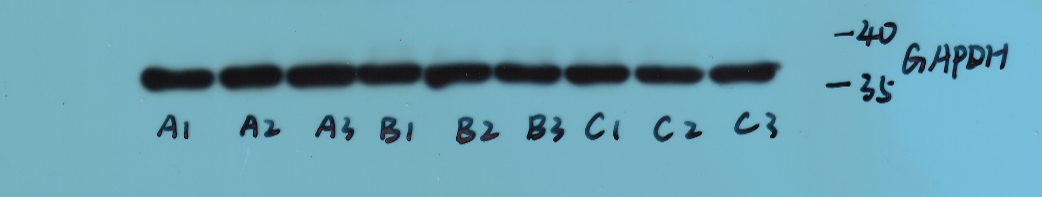


Fig4. shows the proteins expression of fibrotic markers and Smad2/3 proteins expression after cutting membrane at molecular weight 170kDa, 70~55KDa, 55~40KDa and 40~35KDa for Sirt2 (36,42kDa), fibronectin (280KDa), α-SMA (42KDa), p-smad2/smad2 (60KDa), p-smad3/smad3 (52KDa) and GAPDH (36KDa).
