# Supplementary material for: Inhibition of Sirt2 Alleviates Fibroblasts Activation and Pulmonary Fibrosis via Smad2/3 Pathway
Source: Front Pharmacol. 2021 Dec 1;12:756131. doi: 10.3389/fphar.2021.756131 (PMC8672210; doi:10.3389/fphar.2021.756131)
Supplement: Supplementary file 1 [file DataSheet1.zip › original data of sirt2/western blot of cell study.docx]

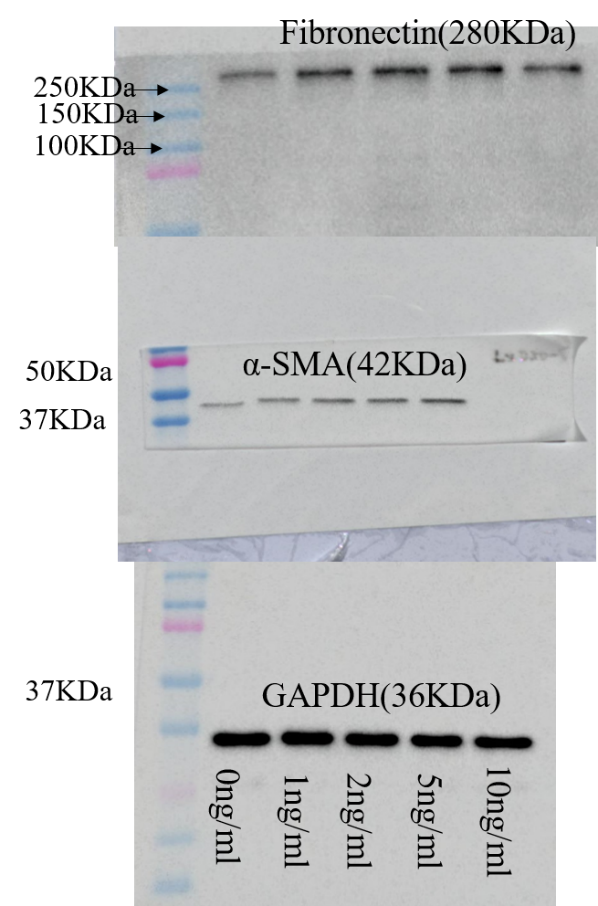

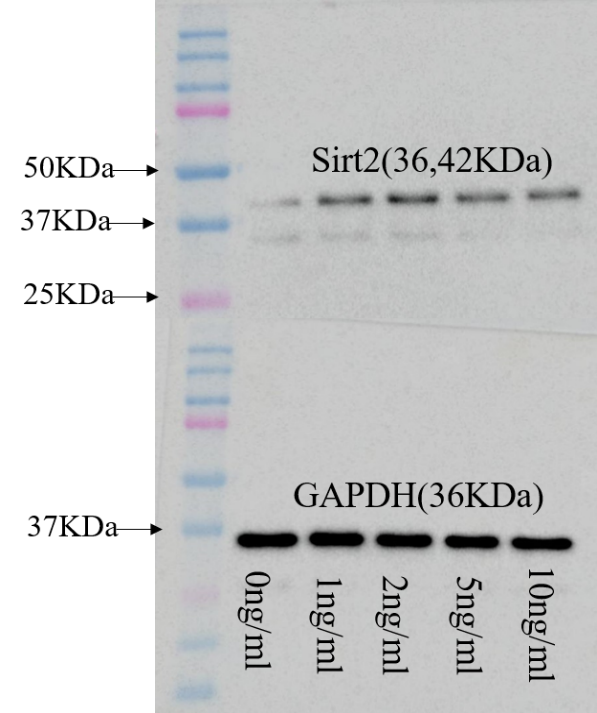


Fig.1 shows expression of fibronectin (280KDa), α-SMA (42KDa), Sirt2 (36.42KDa) and GAPDH (36KDa) in MRC-5 treated with 0 (control), 1, 2, 5 and 10 ng/ml TGF-β1 for 24 h (A whole membrane).


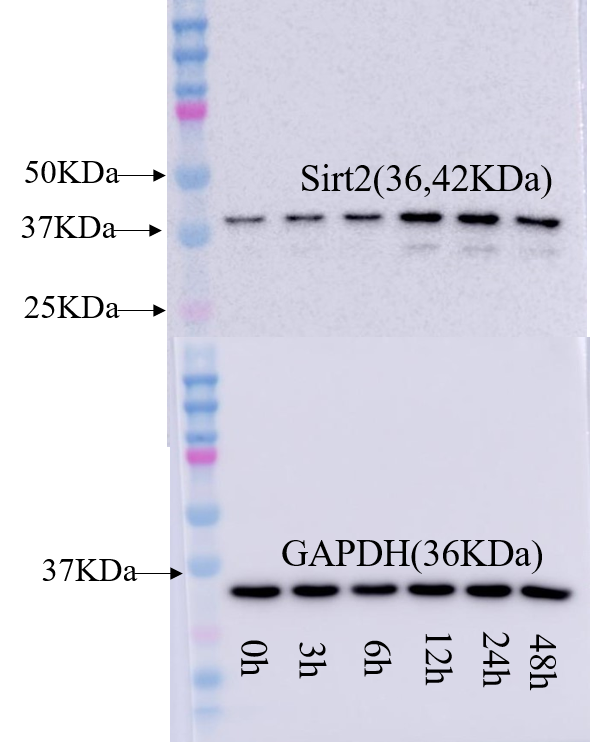


Fig2 shows expression of Sirt2 (36.42KDa) and GAPDH (36KDa) in MRC-5 after 2ng/ml TGF-β1 exposure for 0, 3, 6, 12, 24, and 48 h (A whole membrane).


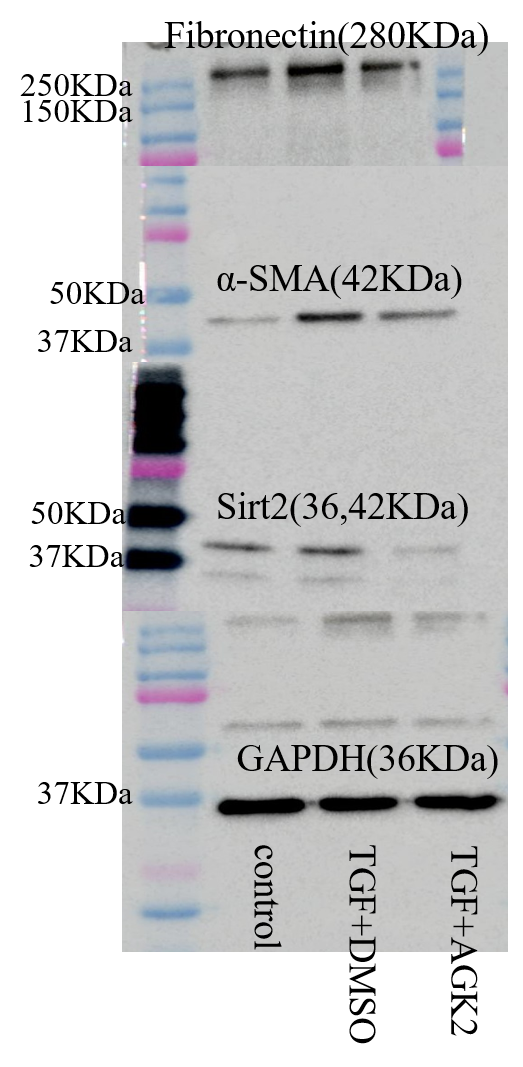


Fig.3 shows expression of fibronectin (280KDa), α-SMA (42KDa), Sirt2 (36.42KDa) and GAPDH (36KDa) in MRC-5 cells pretreated with 2ng/ml TGF-β1 for 24 hours and then 10μM AGK2 or DMSO for 24 hours in the presence of TGF-β1 (A whole membrane).


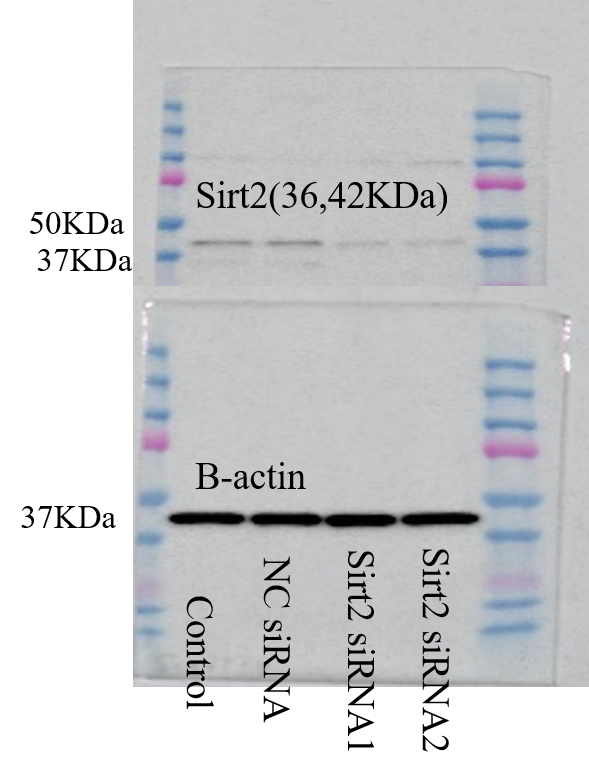


Fig4 shows expression of Sirt2 (36.42KDa) and GAPDH (36KDa) in MRC-5 cells were transfected with NC siRNA or Sirt2 siRNA for 24 hours (A whole membrane).


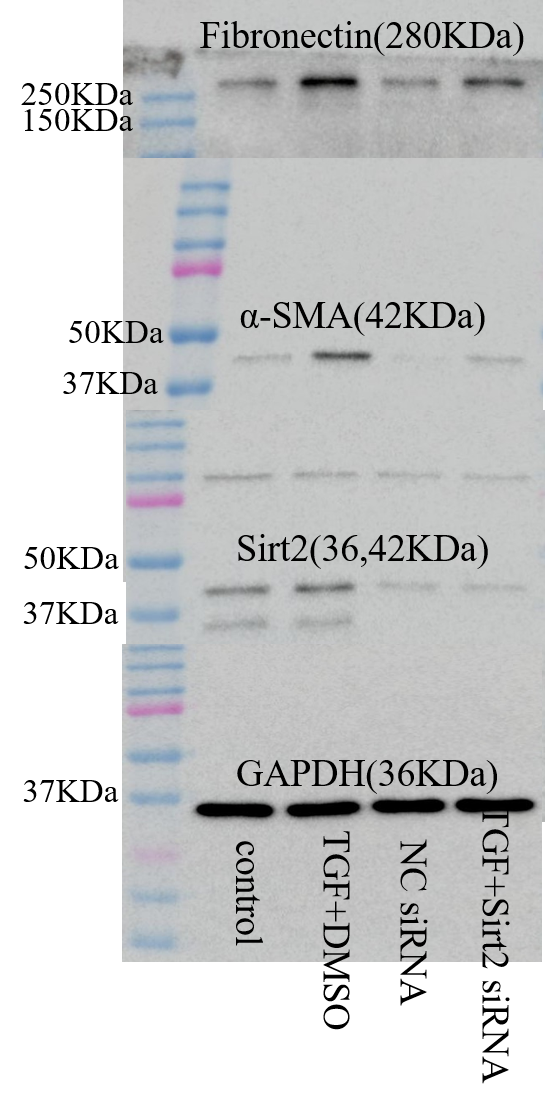

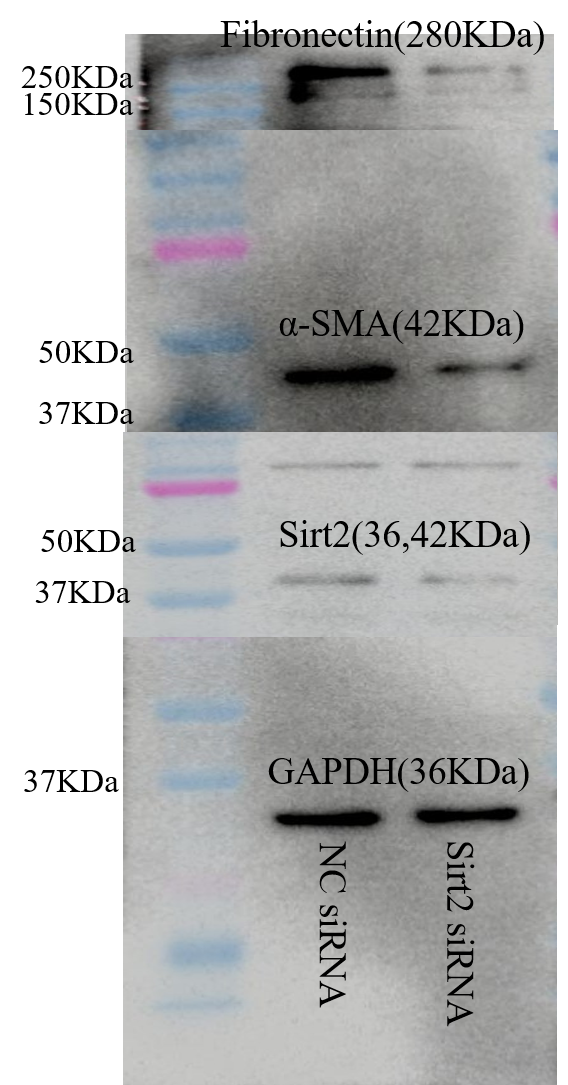


Fig5 shows expression of fibronectin (280KDa), α-SMA (42KDa), Sirt2 (36.42KDa) and GAPDH (36KDa) in MRC-5 cells transfected with NC siRNA or Sirt2 siRNA for 24 hours in the absence or presence of 2ng/ml TGF-β1 (Left); the expression of fibronectin (280KDa), α-SMA (42KDa), Sirt2 (36.42KDa) and GAPDH (36KDa) in IPF lung fibroblasts treated with NC siRNA or Sirt2 siRNA for 24 hours (Right) (A whole membrane).


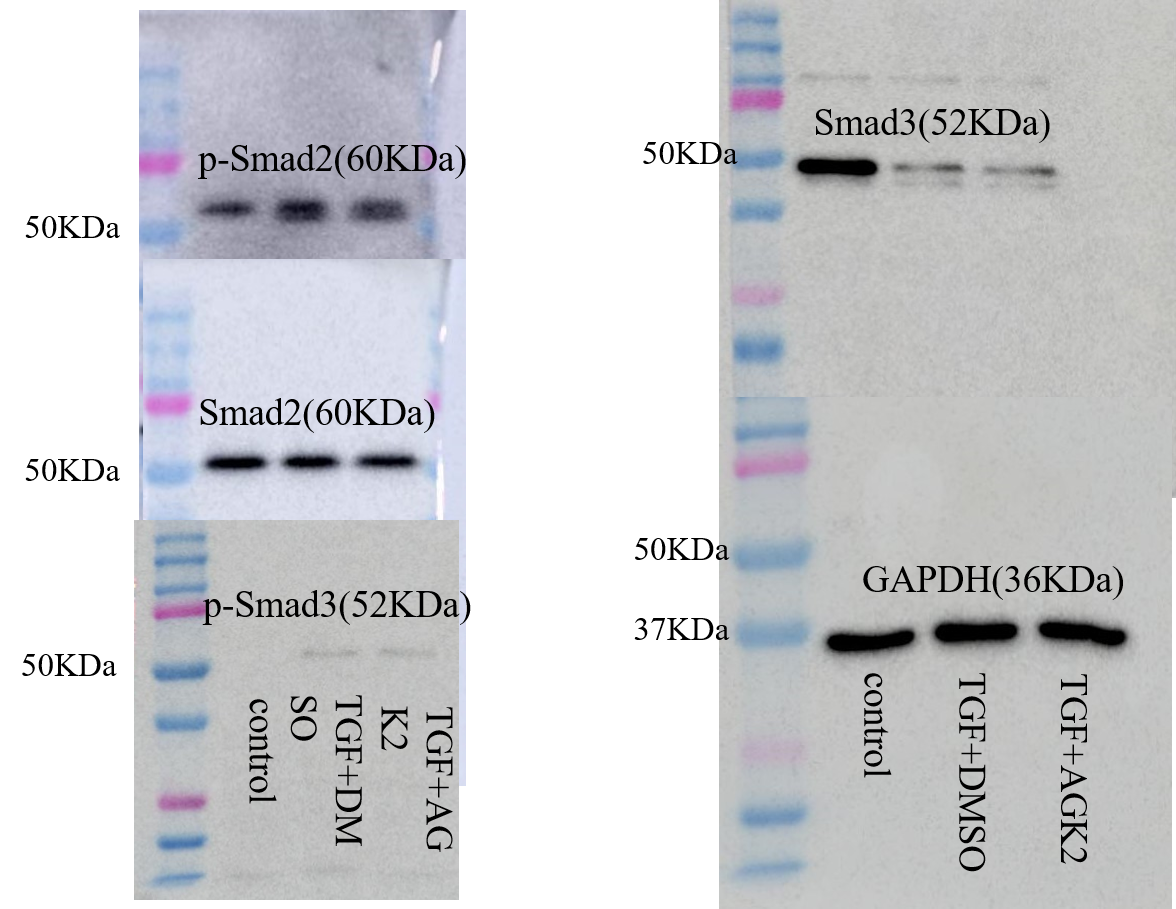


Fig 6 shows expression of p-Smad2/Smad2 (60KDa), p-Smad3/Smad3 (52KDa) and GAPDH (36KDa) by Western blot in MRC-5 cells pretreated with 2ng/ml TGF-β1 for 24 hours and then 10μM AGK2 or DMSO for 24 hours in the presence of TGF-β1 (A whole membrane).


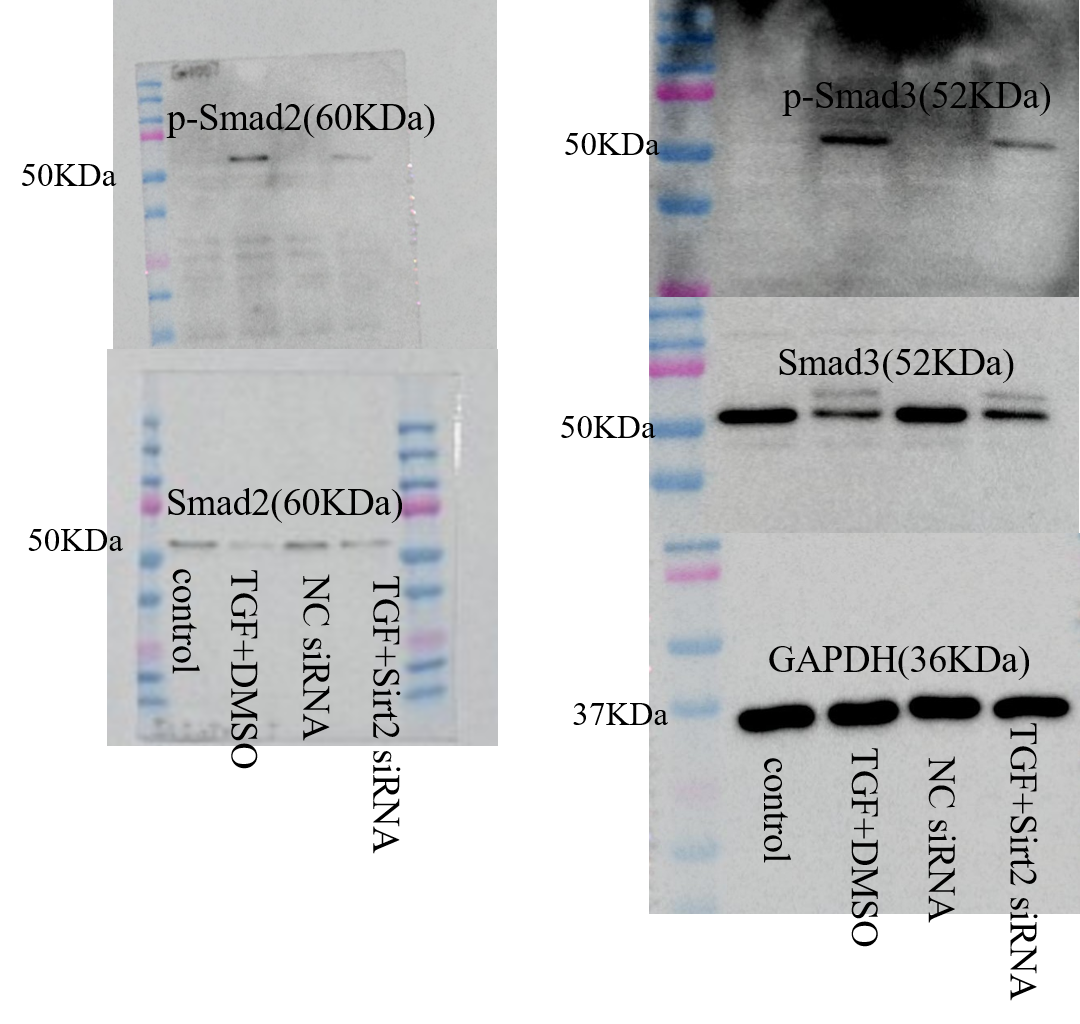


Fig7 shows protein expression of p-Smad2/Smad2 (60KDa), p-Smad3/Smad3 (52KDa) and GAPDH (36KDa) by Western blot in MRC-5 cells transfected with NC siRNA or Sirt2 siRNA for 24 hours in the absence or presence of 2ng/ml TGF-β1 (A whole membrane).


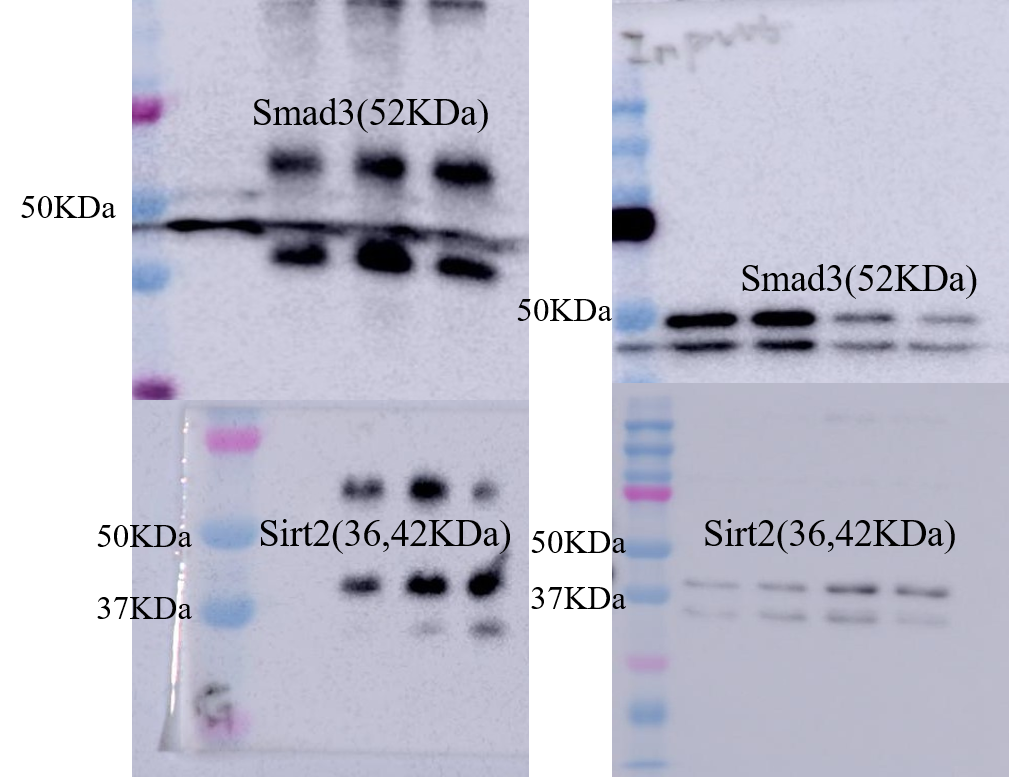


Fig8 shows protein expression of Smad3 and Sirt2.
